# An ORFeome of rice E3 ubiquitin ligases for global analysis of the ubiquitination interactome

**DOI:** 10.1186/s13059-022-02717-8

**Published:** 2022-07-11

**Authors:** Ruyi Wang, Xiaoman You, Chongyang Zhang, Hong Fang, Min Wang, Fan Zhang, Houxiang Kang, Xiao Xu, Zheng Liu, Jiyang Wang, Qingzhen Zhao, Xuli Wang, Zeyun Hao, Feng He, Hui Tao, Debao Wang, Jisong Wang, Liang Fang, Mengchao Qin, Tianxiao Zhao, Pingping Zhang, Hefei Xing, Yunping Xiao, Wende Liu, Qi Xie, Guo-Liang Wang, Yuese Ning

**Affiliations:** 1 State Key Laboratory for Biology of Plant Diseases and Insect Pests, Institute of Plant Protection, Chinese Academy of Agricultural Sciences, Beijing, 100193 China; 2 Department of Plant Pathology, The Ohio State University, Columbus, OH 43210 USA; 3 State Key Laboratory of Plant Genomics, National Center for Plant Gene Research, Institute of Genetics and Developmental Biology, Chinese Academy of Sciences, Beijing, 100101 China; 4 School of Life Sciences, Liaocheng University, Liaocheng, 252000 China; 5 OE Biotech Co., Ltd, Shanghai, 201112 China

**Keywords:** E3 ligase, Ubiquitination, ORFeome, Interactome, Proteomic, Rice

## Abstract

**Background:**

Ubiquitination is essential for many cellular processes in eukaryotes, including 26S proteasome-dependent protein degradation, cell cycle progression, transcriptional regulation, and signal transduction. Although numerous ubiquitinated proteins have been empirically identified, their cognate ubiquitin E3 ligases remain largely unknown.

**Results:**

Here, we generate a complete ubiquitin E3 ligase-encoding open reading frames (UbE3-ORFeome) library containing 98.94% of the 1515 E3 ligase genes in the rice (*Oryza sativa* L*.*) genome. In the test screens with four known ubiquitinated proteins, we identify both known and new E3s. The interaction and degradation between several E3s and their substrates are confirmed in vitro and in vivo. In addition, we identify the F-box E3 ligase OsFBK16 as a hub-interacting protein of the phenylalanine ammonia lyase family OsPAL1–OsPAL7. We demonstrate that OsFBK16 promotes the degradation of OsPAL1, OsPAL5, and OsPAL6. Remarkably, we find that overexpression of *OsPAL1* or *OsPAL6* as well as loss-of-function of *OsFBK16* in rice displayed enhanced blast resistance, indicating that OsFBK16 degrades OsPALs to negatively regulate rice immunity.

**Conclusions:**

The rice UbE3-ORFeome is the first complete E3 ligase library in plants and represents a powerful proteomic resource for rapid identification of the cognate E3 ligases of ubiquitinated proteins and establishment of functional E3–substrate interactome in plants.

**Supplementary Information:**

The online version contains supplementary material available at 10.1186/s13059-022-02717-8.

## Background

Protein interactome analysis enables the comprehensive identification of protein complexes and protein modifications, thus helping us to understand specific biological mechanisms, elucidate cellular functions, and decipher genotype–phenotype relationships [1, 2]. The yeast two-hybrid (Y2H) system has been widely used to analyze direct protein–protein interactions and to provide high-quality binary interactomes [3]. It has been used to develop high-throughput human binary protein–protein interactome maps, from the first-generation maps comprising 2754 pairwise interactions in 2005 [4, 5] to the third-generation maps comprising 53,000 pairwise interactions in 2020 [1].

Protein ubiquitination is required for a plethora of cellular processes in eukaryotes, including proteasome-dependent protein degradation, cell cycle progression, transcriptional regulation, and signal transduction [6] and is sequentially catalyzed by three different types of enzymes: the ubiquitin-activating enzyme E1, the ubiquitin-conjugating enzyme E2, and the ubiquitin ligase E3. E1 catalyzes the ATP-dependent activation of ubiquitin and the formation of a thioester bond with ubiquitin. E2 binds to the activated ubiquitin and transfers it to its substrate via an E3 ligase [6]. E3 ligases, the most heterogeneous of these enzymes, mediate substrate specificity. Their direct physical interactions with substrates determine the modification mode of the substrates. E3 ligases can be divided into single- and multi-subunit types. The single-subunit type includes HECT (homologous to the E6AP carboxyl terminus), RING (really interesting new gene), and U-box, while the multi-subunit type includes SCF (SKP1–CUL1–F-box), CUL3–BTB, CUL4–DDB1–DWD, and APC/C complex (anaphase-promoting complex/cyclosome), in which F-box, BTB, DWD, and APC co-activator subunits determine substrate specificity [7]. The identification of E3 ligases and their substrates is critical for understanding the protein ubiquitination mediated biological processes.

In the past decade, significant progress has been made in identifying ubiquitinated proteins in animals using a monoclonal antibody that specially recognizes the putative ubiquitination sites diglycine-modified lysines (K-Ɛ-GG) [8–10]. Using this antibody, over 20,000 distinct endogenous ubiquitination sites were identified in human cells [11], and 1543 putative ubiquitinated proteins were identified in rice (*Oryza sativa* L*.*) leaves with and without treatment with inducers of plant defense responses [12, 13]. Recently, over 63,000 unique ubiquitination sites on 9200 proteins were identified using the UbiSite antibody, which recognizes the 13 C-terminal amino acids of ubiquitin-specific branched peptides [14]. These approaches have led to the identification of a huge number of putative ubiquitinated proteins in both animals and plants; however, their cognate E3s remain largely unknown.

Plant genomes encode approximately 1500 E3s; this family has expanded by more than two-fold compared to E3s in mammals and other species [6, 15]. This observation suggests that E3 ligases may be involved in regulating many more biological processes in plants than in other species. Although Y2H screening has been widely used to identify E3–substrate pairs in plants, the efficiency of screening E3 ligase genes is low for several reasons. First, two-thirds of the clones from a conventional cDNA library are not amenable to fusion in frame with the N-terminal GAL4 activation domain required to validate interactions [16]. Second, some E3 ligase genes are expressed only in specific tissues or developmental stages or under some stress conditions [17]. Therefore, a complete E3 ubiquitin ORFeome library is essential for analyzing ubiquitination interactome in plants.

Rice is an important food crop and a model monocot plant [18]. In the current study, a ubiquitin E3 ligase gene (UbE3) library covering 98.94% of the 1515 E3 ligase genes in rice was generated. In addition to the known E3s of four substrates, several new E3s were identified by using the UbE3 library. These interactions and substrate degradation were confirmed by in vitro and in vivo assays. Furthermore, when the phenylalanine ammonia lyases OsPAL1–OsPAL9 were used as baits, only the F-box-type E3 ligase OsFBK16 interacted with OsPAL1–OsPAL7*.* We further verified that OsFBK16 degrades OsPAL1, OsPAL5, and OsPAL6 in vivo and demonstrated that overexpression of *OsPAL1* and *OsPAL6* in rice as well as loss-of-function of *OsFBK16* enhanced rice blast disease resistance. Thus, our UbE3 library provides a powerful proteomic resource for the global identification of E3 ligases and analysis of ubiquitination interactome and biological networks in plants.

## Results

### Putative ubiquitinated proteins in rice and annotation of the ubiquitin E3 ligases

We previously identified 1543 proteins containing ubiquitinated sites in two studies [12, 13]. In addition, 1163 and 895 ubiquitinated proteins were recently isolated from germinating rice seeds and young panicles, respectively [19, 20]. These studies led to the identification of 2362 ubiquitinated proteins (Additional file 1: Fig. S1a; Additional file 2: Table S1). Gene Ontology (GO) analysis revealed that these proteins are mainly involved in the nucleic acid metabolic process, reproductive system development, shoot system development, flower development, cell growth, response to external stimulus, and secondary metabolic processes (Additional file 1: Fig. S1b), suggesting that ubiquitinated proteins may play critical roles in plant growth, development, and stress response. In addition, we searched the literature and found that previous studies have characterized the relationships between at least 57 substrates and their E3s (Additional file 1: Fig. S1c and Additional file 3: Table S2); however, only eight of the abovementioned 2362 ubiquitinated proteins were revealed cognate E3s (Additional file 3: Table S2). Altogether, 2411 genes encoding putative ubiquitinated proteins have been identified in rice (Additional file 1: Fig. S1d), accounting for approximately 7.5% of the rice genome (~ 32,000 genes) [21]. Among these, the cognate ubiquitin E3 ligases of only 2.36% have been identified (Additional file 3: Table S2).

**Fig. 1:**
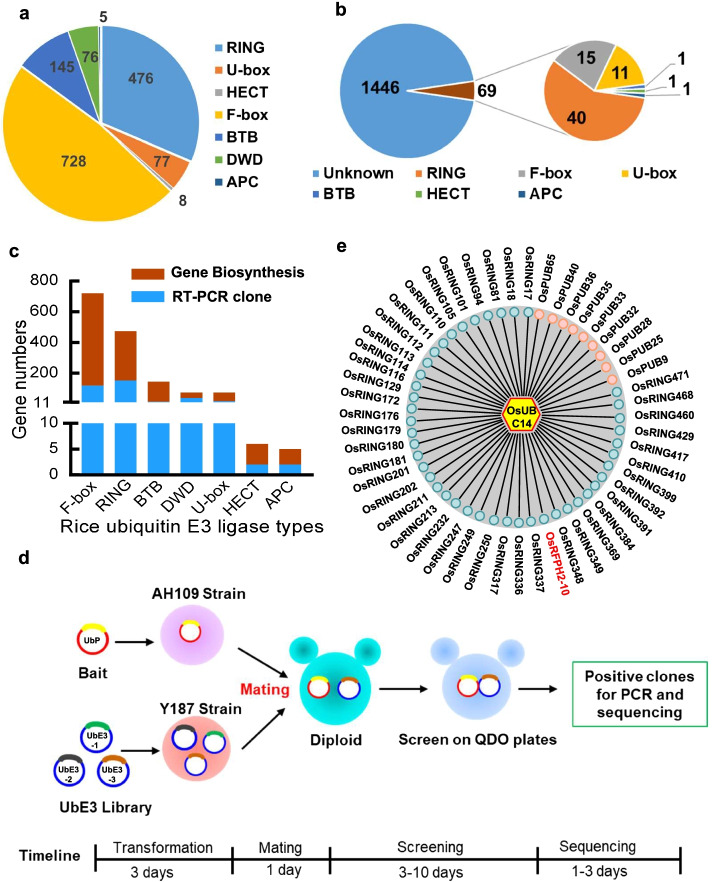
Construction of a ubiquitin E3 ligase-encoding ORFeome library. **a** Number of different types of E3 ligase genes in the rice genome. **b** Functionally characterized E3 ligase genes (69) in rice. **c** Number of synthesized and RT-PCR amplified E3 ligase genes. The *Y*-axis represents the number of synthesized and RT-PCR amplified genes. **d** Flowchart and timeline for Y2H screening of E3 ligases. The bait is inserted into the pGBKT7 (BD) vector and transformed into yeast strain AH109. The E3 ubiquitin ligase library is transformed into yeast strain Y187. **e** Identification of multiple E3 ligases from the rice E3 ligase library using OsUBC14 as the bait

To identify ubiquitin E3 ligases of ubiquitinated proteins, we comprehensively analyzed rice E3 genes based on the sequences in the Nipponbare genome from the MSU Rice Genome Annotation Project Release 7 data (RGAP 7, http://rice.plantbiology.msu.edu/). The number of different types of E3s varies greatly. Eight genes encode HECT-type E3s (OsHECT1–OsHECT8) [22], five encode APC-type E3s (OsCDC20-1, OsCDC20-2, OsCDC20-3, OsCCS52A1/TE/TAD1, OsCCS52B) [23], and 476 encode RING-type E3s [24] (OsRING1–OsRING476) (Additional file 4: Table S3). Among RING-type genes, two pairs are duplicated and share 100% nucleotide sequence identity (*OsRING62* and *OsRING66*; *OsRING202* and *OsRING203*). A total of 728 genes encode F-box-type E3s, including two duplicated genes (*OsFBX466* and *OsFBX55*; *OsFBO24* and *OsFBX481*) [25–27]. We kept the original gene names from RGAP 7 and provided names for previously unidentified genes (Additional file 4: Table S3). In addition, we identified genes encoding 77 putative U-box proteins (OsPUBs) (OsPUB1–OsPUB77) [28], 145 potential BTB-type E3s (with their original names) [29], and 76 DWD motif-containing proteins (named OsDWD1–OsDWD76 based on the results of amino acid sequence alignment and phylogenetic analysis [30]) (Fig. 1a; Additional file 4: Table S3)*.* In total, we identified 1515 E3 genes, which are randomly distributed on 12 rice chromosomes (Fig. 1a and Additional file 1: Fig. S2). It is worth noting that only 69 E3 genes have been functionally characterized (Fig. 1b and Additional file 3: Table S2). Among these, 40 encode RING-type E3s, 15 encode F-box-type E3s, and 11 encode U-box-type E3s (Fig. 1b), accounting for approximately 4.55% of total E3s. These results imply that the functions and substrates of approximately 95.45% of rice E3 genes remain to be elucidated.

### Construction of a high-quality UbE3 yeast library using PCR amplified and synthesized full-length cDNAs

To develop an efficient system to identify potential E3 ligases of substrates, we tried to construct a library that included all of the rice E3 genes. The sequences of the 1515 E3 ligase genes were downloaded from RGAP 7 and used for primer design. To exclude the possibility of the existence of pseudogenes, we searched all of the E3 genes in the Plant Public RNA-seq Database (PPRD, http://ipf.sustech.edu.cn/pub/plantrna/), which integrated all publicly available RNA-seq rice libraries (11,726) [31]. The results revealed that all the E3 genes were expressed (Additional file 5, Table S4). Total RNA was extracted from the leaves of rice cultivar Nipponbare seedlings, followed by reverse transcription to obtain cDNA for gene amplification. The amplified PCR fragments were cloned into the pGADT7 vector. After two rounds of PCR amplification and sequencing, 344 ORFs were obtained, accounting for only 22.71% of an entire set of UbE3 genes (Fig. 1c). Perhaps, only a small proportion of these genes were successfully cloned using the PCR method due to the low abundance of transcripts in seedling tissue. Alternatively, perhaps the complex structures of some genes inhibited the amplification of full-length ORFs. This low cloning efficiency may also explain why only a few E3 genes were identified from the conventional rice cDNA library.

In order to build a complete library including all E3 genes, we synthesized the remaining UbE3 genes based on the sequences from RGAP 7. We synthesized 1,308,698 bp of sequence and obtained the full-length ORFs for 1155 genes. The synthesized ORFs encode 131 BTB- and 601 F-box-type E3s, accounting for 90.34% and 82.55% of their respective E3 types (Fig. 1c; Additional file 1: Fig. S3). These results indicate that large-scale gene synthesis is required in order to clone UbE3 genes in rice. Despite our best efforts, 16 genes were not successfully synthesized due to their huge sizes. For instance, *OsHECT6* is 10,944 bp long. In total, we obtained 1499 clones and constructed a UbE3 ORFeome library, accounting for 98.94% of the E3 ligase genes in the rice genome (Fig. 1c).

To prepare the library, we mixed equal amounts of 1499 individual plasmids and used them to transform yeast strain Y187. This step was completed in 3 days. We aliquoted the transformed yeast cells into small tubes (1 mL) and stored them at -80 °C. We mated yeast strain AH109 carrying the pGBKT7-bait plasmid with the strain Y187 carrying the pGADT7-UbE3 library and incubated them for 20–24 h until a 3-lobed structure appeared. We spread the resuspended sediment onto the SD-Leu-Trp-His-Ade plates and incubated the cultures for 3–10 days until yeast cells grew on the plates and subjected them to PCR and sequencing (1–3 days) (Fig. 1d). The entire screening procedure for one bait took approximately 2 weeks.

Since E2s directly interact with E3s, we chose the E2 OsUBC14 (OsUBC5a), which negatively regulates cell death and immunity in rice, as a test bait [32]. In the mating between AH109 carrying pGBKT7-OsUBC14 and Y187 carrying pGADT7-UbE3, 52 candidate E3s were identified, including 43 RING- and 9 U-box-type E3s (Fig. 1e; Additional file 1: Fig. S4; Additional file 6: Table S5). Among the identified E3s, OsRFPH2-10 is a positive regulator of rice immunity [33]. These results suggest that the UbE3 library is highly efficient for identifying E3 ligases.

### Ubiquitination and degradation of OsSKIPa by two RING-type E3 ligases

**Fig. 2:**
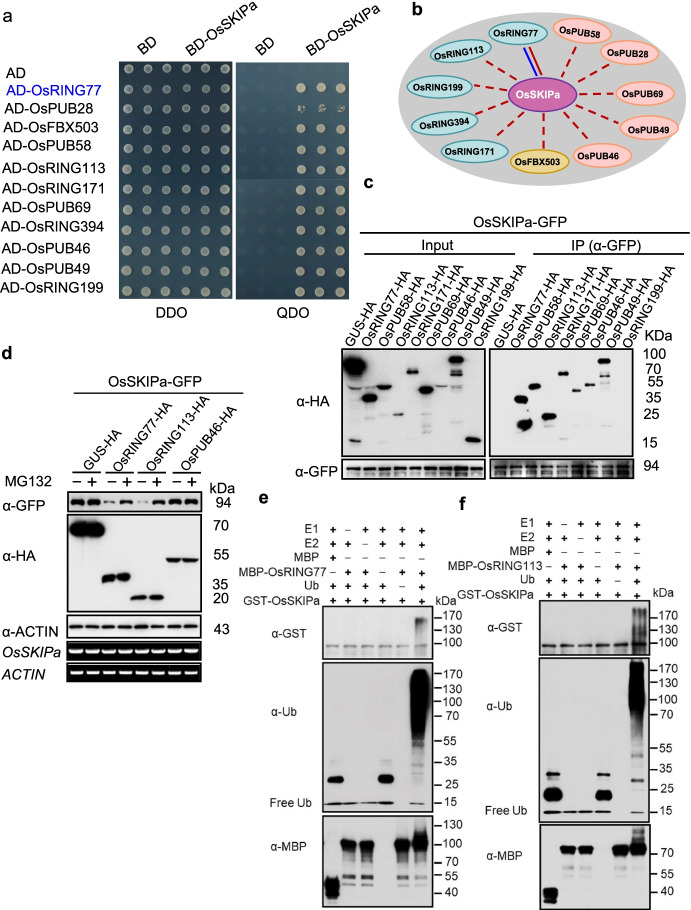
Ubiquitination and degradation of OsSKIPa by two RING-type E3 ligases. **a** Confirmation of the interactions between OsSKIPa and 11 E3 ligases in yeast. Yeast strain AH109 containing BD-OsSKIPa was mated with yeast strain Y187 containing the E3 ubiquitin ligase library. OsRING77 (indicated in blue) was identified in a previous study. DDO, SD-Leu-Trp, QDO, SD-Leu-Trp-His-Ade. **b** Three types of E3 interactors with OsSKIPa. The pink color line represents the identified interaction using the UbE3 library, and the blue color line represents the literature-reported interaction. **c** Co-IP assay of OsSKIPa with eight candidate E3 ligases in planta. *N. benthamiana* leaves were agro-infiltrated with the indicated plasmid combinations. Proteins were extracted and immunoprecipitated with anti-GFP/Agarose beads. Immunoblot detection was performed using anti-GFP or anti-HA antibody as indicated. **d** Degradation assay of OsSKIPa by three E3 ligases (OsRING77, OsRING113, and OsPUB46) with or without 50 μM MG132. *N*. *benthamiana* leaves were agro-infiltrated with plasmids harboring *OsSKIPa-GFP* and three candidate E3 ligases fused with HA tag. GUS-HA was used as a negative control. The protein abundance of OsSKIPa and the candidate E3 ligases was detected by immunoblotting using anti-GFP or anti-HA antibody, respectively. ACTIN was used as an internal control for protein loading. *OsSKIPa* transcript level was measured by RT-PCR, and *ACTIN* was used as the internal control. **e** Ubiquitination assay of OsSKIPa by OsRING77 in vitro. GST-OsSKIPa protein purified from *E. coli* was incubated with MBP-OsRING77 and E1, E2, Ub, and ATP in the reactions. Immunoblot analysis was performed with anti-ubiquitin, anti-MBP, or anti-GST antibody. **f** Ubiquitination assay of OsSKIPa by OsRING113 in vitro. GST-OsSKIPa protein purified from *E. coli* was incubated with MBP-OsRING113 and E1, E2, Ub, and ATP in the reactions. Immunoblot analysis was performed with anti-ubiquitin, anti-MBP, or anti-GST antibody.

As 69 functionally characterized E3 genes were included in the library (Fig. 1b), we chose several substrates with different types of cognate E3s as baits to test the efficiency of the UbE3 library. The first bait protein was the Ski-interacting protein OsSKIPa, which is a positive regulator of plant growth and drought resistance and a putative substrate of RING-type E3 ligases [34]. A total of 51 positive clones were identified in the screen, which harbored 11 unique interactors (Additional file 6: Table S5), including five RING-type E3s (OsRING77, OsRING113, OsRING171, OsRING394, OsRING199), five U-box-type E3s (OsPUB28, OsPUB58, OsPUB69, OsPUB46, OsPUB49), and one F-box-type E3 (OsFBX503) (Fig. 2a, b). Interestingly, among the interactors, OsRING77 was identified in a previous Y2H screen from a rice cDNA library using OsSKIPa as the bait [34]. To validate their interactions in planta, we performed a co-immunoprecipitation (Co-IP) assay using eight candidate E3 ligases (4 RING- and 4 U-box-type E3 ligases). Immunoblot analysis indicated that seven candidates interacted with OsSKIPa, while OsRING199 and the negative control GUS-HA did not (Fig. 2c). These results demonstrate that OsSKIPa directly interacts with multiple E3 ligases *in planta*.

To investigate whether these candidate interactors are functional E3 ligase, we performed in vitro protein ubiquitination assay. Compared to the negative controls, high-molecular-weight smear bands were observed by immunoblot analysis with anti-ubiquitin antibody using the combination of MBP-OsRING77, MBP-OsRING113, MBP-OsPUB28, MBP-OsPUB46, MBP-OsPUB49, or MBP-OsPUB69 fusion protein with E1, E2, ubiquitin, and ATP (Additional file 1: Fig. S5a-f), while OsRING171 and OsPUB58 not, demonstrating that OsRING77, OsRING113, OsPUB28, OsPUB46 OsPUB49, and OsPUB69 exhibit strong E3 ligase activity. To examine whether these E3 ligases promote the degradation of OsSKIPa, we monitored the protein stability of OsSKIPa when it was co-expressed with three E3s (2 RING- and 1 U-box-type E3s) separately in *Nicotiana benthamiana* leaves in the presence or absence of the 26S proteasome inhibitor MG132 (Fig. 2d). Compared to the negative control GUS-HA, significant degradation was observed when OsRING77 or OsRING113 was co-expressed with OsSKIPa-GFP, but not when OsPUB46 was co-expressed with OsSKIPa-GFP, although OsPUB46 also interacted with OsSKIPa in Y2H and Co-IP assays. The addition of MG132 strongly inhibited the degradation of OsSKIPa (Fig. 2d). These results suggest that OsRING77 and OsRING113 promote the 26S proteasome-dependent degradation of OsSKIPa.

To test the idea that the degradation of OsSKIPa was directly due to E3 ligase-mediated polyubiquitination, purified GFP-OsSKIPa was added to the above in vitro ubiquitination reaction containing MBP-OsRING77, MBP-OsRING113, or MBP-OsPUB46. A significant polyubiquitination signal was detected with anti-GST antibody in the presence of OsRING77 or OsRING113 but not OsPUB46 (Fig. 2e, f; Additional file 1: Fig. S6), indicating that both OsRING77 and OsRING113 can ubiquitinate OsSKIPa in vitro. These results demonstrate that OsSKIPa is a substrate of both RING-type E3 ligases and is degraded in the presence of these E3 ligases.

### Ubiquitination and degradation of OsNRPD1a by both U-box- and RING-type E3 ligases

**Fig. 3:**
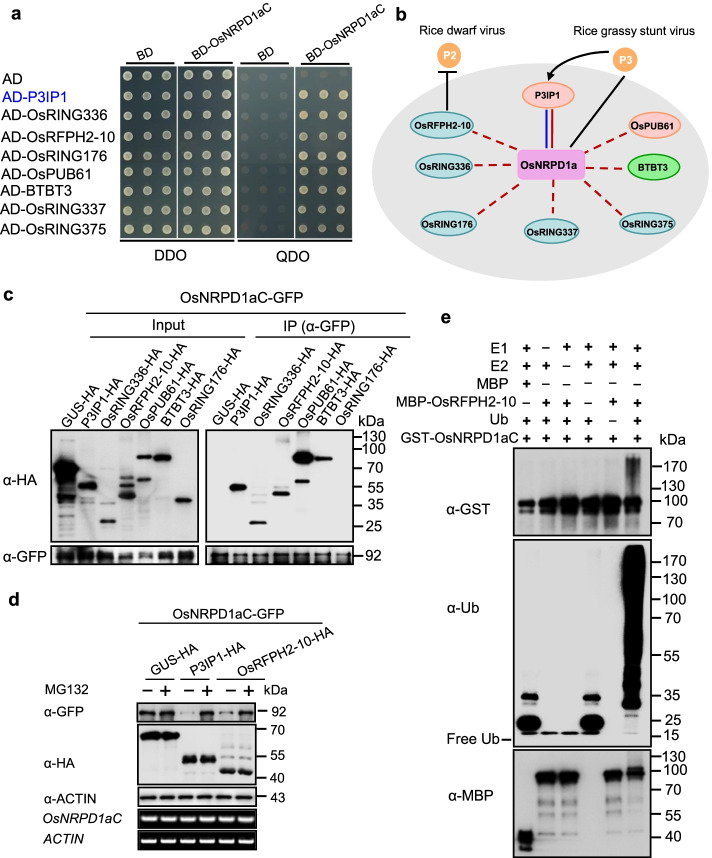
Ubiquitination and degradation of OsNRPD1a by both U-box- and RING-type E3 ligases. **a** Confirmation of the interaction between OsNRPD1aC and eight candidate E3 ligases in yeast. P3IP1 (indicated in blue) was identified as the cognate E3 of OsNRPD1a in a previous study. DDO, SD-Leu-Trp, QDO, SD-Leu-Trp-His-Ade. **b** Names and functions of the E3 ligases that interacted with OsNRPD1aC. The pink color line represents the identified interaction using the UbE3 library, and the blue color line represents the literature-reported interaction. **c** Co-IP assay of OsNRPD1aC with six candidate E3 ligases. *N. benthamiana* leaves were agro-infiltrated with the indicated plasmid combinations. Total protein was extracted and immunoprecipitated with anti-GFP/Agarose beads. Immunoblot detection was performed using anti-GFP or anti-HA antibody as indicated. **d** Degradation assay of OsNRPD1aC by P3IP1 and OsRFPH2-10 with or without 50 μM MG132 treatment. *N*. *benthamiana* leaves were agro-infiltrated with plasmids harboring *OsNRPD1aC-GFP* and two E3 ligase genes fused with HA tag. GUS-HA was used as a negative control. The protein abundance of OsNRPD1aC and the two E3 ligases were detected by immunoblotting using anti-GFP or anti-HA antibody, respectively. ACTIN was used as an internal control. *OsNRPD1aC* transcript level was measured by RT-PCR, and *ACTIN* was used as the internal control. **e** Ubiquitination assay of OsNRPD1aC by OsRFPH2-10. OsNRPD1aC protein purified from *E. coli* was incubated with MBP-OsRFPH2-10 and E1, E2, Ub, and ATP in the reactions. Immunoblot analysis was performed using anti-ubiquitin, anti-MBP, or anti-GST antibody

To determine whether U-box-type E3 ligases could be efficiently screened from the UbE3 library, we selected NUCLEAR RNA POLYMERASE D1a (OsNRPD1a) as a test bait; this protein interacts with the U-box-type E3 P3IP1 (OsPUB45) and negatively regulates resistance against rice grassy stunt virus (RGSV) [35]. When the OsNRPD1a C-terminus (OsNRPD1aC) was used as the bait, we obtained 92 positive clones and eight non-redundant E3s (Additional file 6: Table S5). Besides P3IP1, seven other E3 ligases were identified: one U-box-type E3 (OsPUB61), five RING-type E3s (OsRFPH2-10/OsRING344, OsRING336, OsRING176, OsRING337, RING375), and one BTB-type E3 (BTBT3) (Fig. 3a, b). Among these, OsRFPH2-10 was reported to interact with the rice dwarf virus (RDV) P2 protein and promote the degradation of this viral protein [33]. Subsequently, we performed Co-IP assays between six of the eight candidates (three RING-, two U-box-, and one BTB-type E3 ligases) and OsNRPD1aC in *N. benthamiana* leaves. The assays showed that OsPUB61, OsRFPH2-10, OsRING336, and BTBT3 interacted with OsNRPD1aC-GFP individually, as did the positive control P3IP1 (Fig. 3c), indicating that OsNRPD1aC interacts with these E3 ligases in vivo.

We then purified MBP-P3IP1, MBP-OsRFPH2-10, MBP-OsRING176, and MBP-OsRING336 fusion proteins individually and performed E3 ubiquitin ligase activity assays in vitro*.* The assay showed that OsRFPH2-10 and OsRING336, like P3IP1, were active ubiquitin ligases (Additional file 1: Fig. S7a-c). The U-box protein P3IP1 promotes the 26S proteasome-dependent degradation of OsNRPD1a [35]. To investigate whether these novel E3 ligases possess a similar function, we conducted a degradation assay in *N. benthamiana* leaves. The assay revealed that like P3IP1, OsNRPD1aC levels significantly decreased when co-expressed with OsRFPH2-10 compared with the control GUS-HA (Fig. 3d). MG132 treatment strongly prevented the turnover of OsNRPD1aC mediated by either P3IP1 or OsRFPH2-10 (Fig. 3d). These data demonstrate that both P3IP1 and OsRFPH2-10 are likely the cognate E3s of OsNRPD1a in rice.

When we added the GST-OsNRPD1aC fusion protein to the in vitro ubiquitination reaction containing MBP-OsRFPH2-10 protein, prominent high-molecular-weight polyubiquitination bands were detected in the reaction with OsRFPH2-10 (Fig. 3e). These data demonstrate that the RING-type E3 ligase OsRFPH2-10 and the U-box-type E3 ligase P3IP1 can be effectively screened from the UbE3 library using OsNRPD1aC as a bait and that OsRFPH2-10 is a new cognate E3 ligase of OsNRPD1a.

### Identification of BTB-type E3 ligases that interact with NRR and rTGA2.1

**Fig. 4:**
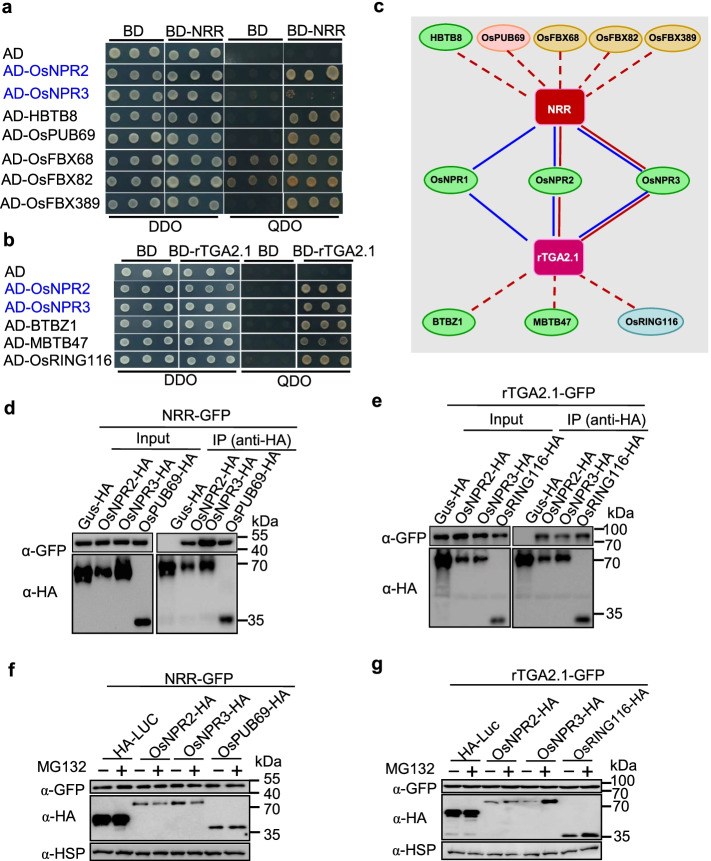
Identification of BTB-type E3 ligases that interact with NRR and rTGA2.1. **a** Confirmation of the interaction between NRR and seven candidate E3 ligases. OsNPR2 and OsNPR3 (labeled in blue) were identified in a previous study. DDO, SD-Leu-Trp, QDO, SD-Leu-Trp-His-Ade. **b** Confirmation of the interaction between rTGA2.1 and five candidate E3 ligases. OsNPR2 and OsNPR3 (labeled in blue) were identified in a previous study. DDO, SD-Leu-Trp, QDO, SD-Leu-Trp-His-Ade. **c** Interaction network among NRR and rTGA2.1 and 11 candidate E3 ligases. The pink color line represents the identified interaction using the UbE3 library, and the blue color line represents the literature-reported interaction. **d**, **e** Co-IP assay of NRR or rTGA2.1 with their candidate E3 ligases in vivo. *N. benthamiana* leaves were agro-infiltrated with the indicated plasmid combinations. Total protein was extracted and immunoprecipitated with anti-HA/Agarose beads. Immunoblot detection was performed using anti-GFP or anti-HA antibody as indicated. **f**, **g** Degradation assay of NRR and rTGA2.1 by their candidate E3 ligases with or without 50 μM MG132 treatment. Plasmids harboring *NRR-GFP* (**f**) or *rTGA2.1-GFP* (**g**) and with their respective candidate E3 ligase genes fused with HA tag were co-expressed in rice protoplasts. GUS-HA was used as a negative control. At 16 h after co-transfection, 50 μM MG132 or an equal volume of DMSO solution was added to the protoplasts. The protoplasts were collected for protein extraction 4 h after MG132 treatment. NRR and rTGA2.1 protein abundance was detected by immunoblotting using an anti-GFP antibody, and E3 ligases were detected by an anti-HA antibody. HSP was used as an internal control

Next, we tested whether BTB-type E3s could be efficiently screened from the UbE3 library. Both NRR and rTGA2.1 interact with BTB-type E3s OsNPR1-3 and negatively regulate resistance against the bacterial pathogen *Xanthomonas oryzae* pv*. Oryzae* [36, 37]. Remarkably, both OsNPR2 and OsNPR3 were identified when either NRR or rTGA2.1 was used as the bait (Fig. 4a–c). We also identified another BTB-type E3 (HBTB8), one U-box-type E3 (OsPUB69), and three F-box-type E3s (OsFBX68, OsFBX82, and OsFBX389) using NRR as the bait. Additionally, two BTB-type E3s (BTBZ1 and MBTB47) and one RING-type E3 (OsRING116) were identified using rTGA2.1 as the bait (Fig. 4a–c; Additional file 6: Table S5). These novel E3s could be important materials for dissecting the NRR- or rTGA2.1-mediated resistance pathway in rice.

To confirm the interactions between the candidate E3 ligases and NRR or rTGA2.1 that were identified in yeast, we performed a Co-IP assay in *N. benthamiana*. NRR-GFP was detected using the anti-GFP antibody in the complexes immunoprecipitated by OsNPR2-HA, OsNPR3-HA, and OsPUB69-HA, but not by GUS-HA (Fig. 4d), suggesting that OsNPR2, OsNPR3, and OsPUB69 interact with NRR in vivo. A similar assay confirmed the interactions between rTGA2.1 and OsNPR2, OsNPR3, or OsRING116 (Fig. 4e). However, the degradation assay and MG132 treatment in *N. benthamiana* leaves failed to detect the turnover of NRR or rTGA2.1 (data not shown). To examine whether this turnover only occurs in rice cells, we co-transfected rice protoplasts with *NRR-GFP* and *OsNPR2-HA*, *OsNPR3-HA*, or *OsPUB69-HA*. No significant change in NRR-GFP was observed compared to the negative control combination of *NRR-GFP* and *HA-LUC*, and MG132 treatment did not facilitate the protein enrichment (Fig. 4f). Similar results were obtained for rTGA2.1-GFP and OsNPR2-HA, OsNPR3-HA, or OsRING116-HA (Fig. 4g). Together, the results suggest that OsNPR2 and OsNPR3 as well as the newly identified E3 ligases may not degrade NRR or rTGA2.1 in *N. benthamiana* leaves and rice protoplasts.

In an in vitro ubiquitination assay, we also failed to observe any protein modifications on rTGA2.1 induced by OsRING116, although OsRING116 displayed strong E3 activity (Additional file 1: Fig. S8a, b). We suspect that NRR and rTGA2.1 might be regulated by other modifications rather than polyubiquitination-dependent degradation. Together, these results demonstrate that the BTB-type E3s could be effectively screened from the UbE3 library and that NRR and rTGA2.1 not only interact with BTB-type E3s, but also with F-box, U-box, and RING-type E3s.

### The F-box E3 ligase OsFBK16 interacts with and degrades OsPALs to negatively regulate blast resistance

**Fig. 5:**
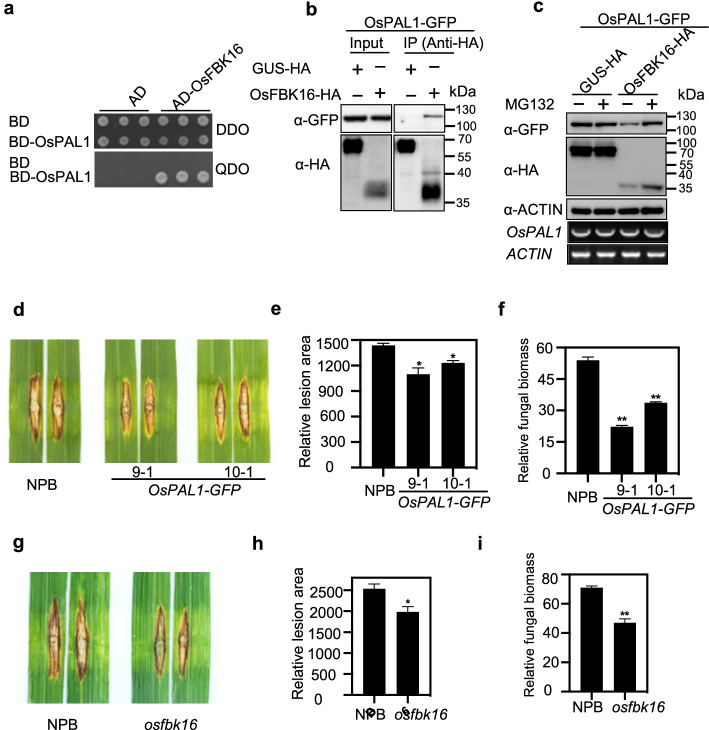
OsFBK16 interacts with and degrades OsPAL1 to modulate rice blast resistance. **a** The AH109 yeast strain containing BD-OsPAL1 mated with the Y187 strain containing the E3 ubiquitin ligase library for screening. DDO, SD-Leu-Trp, QDO, SD-Leu-Trp-His-Ade. **b** Co-IP assay of OsPAL1 with OsFBK16 in vivo. *N. benthamiana* leaves were agro-infiltrated with the indicated plasmid combinations. Total protein was extracted and immunoprecipitated with anti-HA/Agarose beads. Immunoblot detection was performed using anti-GFP or HA antibody as indicated. **c** Degradation assay of OsPAL1 by OsFBK16 in the presence or absence of 50 μM MG132. *N*. *benthamiana* leaves were agro-infiltrated with OsPAL1-GFP and OsFBK16-HA or GUS-HA. Samples were collected, and total protein was extracted. The protein abundance of OsPAL1 was detected by immunoblotting using an anti-GFP antibody, and OsFBK16 and GUS were detected using an anti-HA antibody. ACTIN was used as the internal control. *OsPAL1* transcript levels were measured by RT-PCR, and *ACTIN* was used as the internal control. **d**–**f** Disease symptoms of two representative *OsPAL1-GFP* overexpression lines and NPB seedlings after punch inoculation with the compatible *M. oryzae* isolate RB22. **d** The images were taken 14 days after inoculation. **e** The disease lesion area was measured with the ImageJ software. An asterisk indicates a significant difference between WT and *OsPAL1-GFP* overexpression plants according to Student’s *t* test (**p < 0.01). **f** Relative fungal biomass was calculated by measuring the expression of the *M. oryzae MoPot2* with the DNA-based quantitative PCR assay. An asterisk indicates a significant difference between WT and *OsPAL1-GFP* overexpression plants according to Student’s *t* test (**p < 0.01). **g**–**i** Disease symptoms of the *osfbk16* mutant and NPB seedlings after punch inoculation with RB22. **g** The images were taken 14 days after inoculation. **h** The disease lesion area was measured with the ImageJ software. An asterisk indicates a significant difference between WT and the *osfbk16* mutant according to Student’s *t* test (**p < 0.01). **i** Relative fungal biomass was calculated by measuring the expression of the *M. oryzae MoPot2* with the DNA-based quantitative PCR assay. An asterisk indicates a significant difference between WT and *osfbk16* mutants according to Student’s *t* test (**p < 0.01)

Phenylalanine ammonia-lyase (PAL) catalyzes the first step in the phenylpropanoid biosynthesis pathway. Ubiquitome analyses revealed 17 ubiquitinated sites in OsPAL1 [13]. To investigate which E3 ligase regulates the degradation of OsPAL1, we screened the rice UbE3 library using OsPAL1 as the bait. Surprisingly, sequencing results revealed that 111 of the 124 identified clones contain the same gene encoding F-box-type E3, OsFBK16. The interaction between OsPAL1 and OsFBK16 was confirmed in yeast co-transformation assay and in *N. benthamiana* Co-IP assay, respectively (Fig. 5a, b). We then performed a degradation assay in *N. benthamiana* leaves and found that OsPAL1 protein abundance was reduced when it was co-expressed with OsFBK16-HA but not with the negative control GUS-HA, whereas MG132 treatment strongly inhibited this degradation (Fig. 5c). These results demonstrate that the F-box protein OsFBK16 can promote the degradation of OsPAL1*.*

PAL enzymes have emerged as key players in broad-spectrum disease resistance in rice [38] and overexpression of *OsPAL1* in rice cultivar TP309 enhanced resistance against the *M. oryzae* isolate Zhong1 [39]. We generated *OsPAL1* overexpression lines in the NPB background (Additional file 1: Fig S9a), and the punch inoculation with the *M. oryzae* compatible isolate RB22 showed that *OsPAL1*-OX plants have smaller lesions and less fungal biomass compared with NPB (Fig. 5d–f), consistent with the positive role of *OsPAL1* in blast disease resistance. Because OsFBK16 can promote the degradation of OsPAL1 (Fig. 5c), we speculated that OsFBK16 may negatively regulate rice blast resistance. We subsequently generated the *OsFBK16* knockout mutant by CRISPR/Cas9 in the NPB background (Additional file 1: Fig. S9b). Similar to the *OsPAL1*-OX plants, the *osfbk16* mutant also exhibited smaller disease lesions and less fungal biomass compared with NPB (Fig. 5g–i), demonstrating that OsFBK16 degrades OsPAL1 to negatively regulate rice blast resistance.

**Fig. 6:**
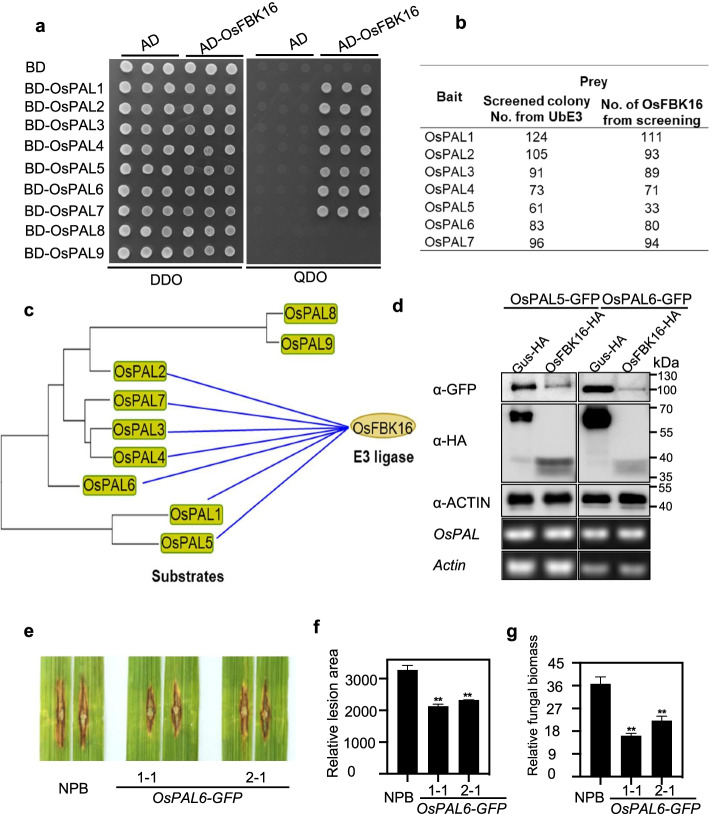
OsFBK16 is the hub interactor of OsPALs and *OsPAL6* confers rice blast resistance. **a** Confirmation of the interaction between OsPAL family members and OsFBK16 in yeast. DDO, SD-Leu-Trp, QDO, and SD-Leu-Trp-His-Ade. **b** Colony number of OsFBK6 screened by OsPAL1-7 from the rice UbE3 library. **c** Interaction between OsPAL family members and E3 ligase OsFBK16. **d** Degradation assay of OsPAL5 and OsPAL6 by OsFBK16 in vivo. *N*. *benthamiana* leaves were agro-infiltrated with *OsPAL5-GFP* or *OsPAL6-GFP* and *OsFBK16-HA* or *GUS-HA*. Samples were collected, and total protein was extracted. The protein abundance of OsPAL5 and OsPAL6 were detected by immunoblotting using an anti-GFP antibody, and OsFBK16 and GUS were detected using an anti-HA antibody. *OsPAL5* and *OsPAL6* transcript levels were measured by RT-PCR, and *ACTIN* was used as the internal control. **e**–**g** Disease symptoms of two representative *OsPAL6-GFP* overexpression lines and NPB seedlings after punch inoculation with RB22. **e** The images were taken 14 days after inoculation. **f** The disease lesion area was measured with the ImageJ software. An asterisk indicates a significant difference between WT and *OsPAL6-GFP* overexpression plants according to Student’s *t* test (**p < 0.01). **g** Relative fungal biomass was calculated by measuring the expression of the *M. oryzae MoPot2* with the DNA-based quantitative PCR assay. An asterisk indicates a significant difference between WT and *OsPAL6-GFP* overexpression plants according to Student’s *t* test (**p < 0.01)

Besides OsPAL1, other PAL proteins also displayed a significant increase in ubiquitination following chitin or flg22 treatment in the ubiquitome analyses [13]. We then screened the rice UbE3 library using eight other OsPALs as baits*.* Remarkably, six of the eight OsPALs (all except OsPAL8 and OsPAL9), i.e., OsPAL2–OsPAL7, convergently interacted with OsFBK16 (Fig. 6a, b and Additional file 6: Table S5). Phylogenetic tree analysis revealed that OsPAL1–OsPAL7 share closer relationships among themselves than OsPAL8 and OsPAL9 (Fig. 6c). We randomly chose OsPAL5 and OsPAL6 to carry out the degradation assay in *N. benthamiana* leaves and found that both OsPAL5 and OsPAL6 protein was decreased when it was co-expressed with OsFBK16 compared with the negative control GUS-HA (Fig. 6d). We confirmed the degradation in rice cells by co-transfecting rice protoplasts with *OsPAL5-GFP/OsPAL6-GFP* and *OsFBK16-HA*, or *GUS-HA* (Additional file 1: Fig. S10). Both in vivo systems revealed that OsPAL5 and OsPAL6 proteins were decreased when co-expression with OsFBK16 (Fig. 6d and Additional file 1: Fig. S10), which is similar to that of OsPAL1. We thus infer OsPAL5 and OsPAL6 may also act as a positive regulator in rice immunity. When overexpressed *OsPAL6* in rice, the plants displayed enhanced disease resistance (Additional file 1: Fig. S11 and Fig. 6e-g), which is similar with the *osfbk16* mutant phenotype, indicating that OsPAL6 has a similar function with OsPAL1 and positively regulates blast resistance. The results from the degradation and disease resistance analyses suggest that the F-box type E3 ligase OsFBK16 targets multiple OsPAL members for degradation to negatively regulate rice immunity. Additionally, these results demonstrate that our rice UbE3 library can be used for the rapid identification of new E3s for functional and biological network analysis.

## Discussion

The identification of E3–substrate pairs is a challenging task due to the reversible nature of ubiquitination, their low affinity, transient interactions, low cellular abundance, and rapid degradation of ubiquitinated substrates [40, 41]. Although various approaches have been developed to define substrates of known E3s, which have led to the identification of > 4600 ubiquitinated proteins in mammals [9, 10] and 2362 ubiquitinated proteins in rice, it is extremely difficult to identify the cognate E3 ligases of these proteins using current methods and resources. In this study, we analyzed ubiquitin E3 ligase-encoding ORFs in rice and re-annotated the genes. All of the E3 genes were expressed in the Plant Public RNA-seq Database, while only 22.71% of them were cloned using the RT-PCR method in our experimental conditions, the small proportion is mainly because of the tissue-specific expression of E3s in leaves. Using RT-PCR cloning and DNA chemical synthesis, we constructed an entire set of rice UbE3-ORFeome, accounting for 98.94% of ubiquitin E3 ligase genes in the rice genome. We generated a yeast library containing equal concentrations of plasmids harboring E3 genes that allowed us to identify all possible E3s of a substrate. Moreover, all the clones in the UbE3 library were sequencing-verified, which began with the start codon and remained in frame with the N-terminal GAL4 AD domain, thus guaranteeing the correct translation of the clones in yeast. The library was transformed into yeast strain Y187, which can easily be mated with a bait yeast strain. The entire E3 ligase screening procedure from yeast strain mating to sequence confirmation of positive clones could be completed within 2 weeks. To the best of our knowledge, this is the first complete E3 ligase library constructed in plants. Combining the approaches of ubiquitination site identification by the K-Ɛ-GG antibody and corresponding E3 ligase screening by the UbE3 library will greatly contribute to illustrate the molecular mechanism of protein ubiquitination.

Using several known ubiquitinated proteins as test baits, we not only successfully identified their known E3s that were reported in previous studies but also novel E3 ligases, thereby expanding the ubiquitination interaction network of these proteins. For example, when the RGSV resistance regulator OsNRPD1aC was used as a bait, in addition to the known U-box-type E3 P3IP1, we also identified seven new E3 ligases. One of these is the RING-type E3 OsRFPH2-10, which was revealed to function as an RDV resistance regulator [33]. Interestingly, we showed that OsRFPH2-10 can polyubiquitinate OsNRPD1a and facilitate its 26S proteasome-dependent degradation. This finding suggests that OsRFPH2-10 might also regulate resistance against RGSV and that OsNRPD1a might function as a hub that modulates immunity to different viruses. Similarly, five new E3 ligases were identified when NRR was used as the bait. These results demonstrate that the E3–substrate relationship in rice is complex and that the systematic analysis of the ubiquitination interactome using our UbE3 library will greatly facilitate the establishment of a ubiquitination interactome in rice.

Conserved proteins and interactomes among different plants contribute to understanding evolutionary relationships among interacting proteins [17]. The roles of orthologs of E3–substrate pairs involved in similar signaling pathways in *Arabidopsis* and rice were recently demonstrated. For instance, both rice *D3* and its ortholog *MAX2* (*MORE AXILLARY GROWTH2*) in *Arabidopsis* encode F-box-type E3 ligases that interact with their homologous repressor proteins D53 and SMXL6/7/8 (SUPPRESSOR OF MAX2 1-Like 6/7/8), respectively, for ubiquitination and 26S proteasome-mediated proteolysis to regulate strigolactone responses [42–44]. In the current study, we found that the F-box-type E3 OsFBK16 interacts with seven of the nine members of the OsPAL family (OsPAL1–OsPAL7) and degrades at least OsPAL1, OsPAL5, and OsPAL6 to negatively regulate rice blast resistance. Intriguingly, interactions and 26S proteasome-mediated degradation also occur between F-box-type E3s AtKFB01/20/50 (orthologs of OsFBK16) and AtPAL1/2/3/4 in *Arabidopsis* [45]. It was reported that *atpal1 atpal2 atpal3 atpal4* quadruple knockout mutants display increased susceptibility to the bacterial pathogen *Pseudomonas syringae* [46]. Based on the disease resistance phenotypes of the overexpression plants of *OsPAL1* or *OsPAL6* and the loss-of-function mutation of *OsFBK16*, it can be speculated that AtKFB01/20/50 may also negatively regulate resistance to bacterial pathogens. These findings suggest that a conserved interaction and regulatory mechanism exist between the PAL–FBK pairs in rice and *Arabidopsis.* On the other hand, the F-box protein ZmFBL41 in maize negatively regulates the resistance to *Rhizoctonia solani* through interacting with and ubiquitinating ZmCAD (cinnamyl alcohol dehydrogenase) for degradation [47]. Interestingly, knocking out of *OsCAD8B*, an ortholog of *ZmCAD* in the rice cultivar ZH11, also reduces resistance to *R. solani*. Using the rice UbE3 library generated in this study, the E3 ligase that can interact with and ubiquitinate OsCAD8B can be easily identified. A recent deep proteomic profiling study co-fractionation mass spectrometry uncovered conserved protein complexes in 13 diverse plant species, including the crop plants, such as rice, maize, soybean, wheat, and the model plant *Arabidopsis* [2]. Due to the availability of the rice UbE3 library, we expect that additional E3–substrate interactions will be revealed rapidly in the near future. Such rice ubiquitination interactome data will be valuable for the establishment of similar networks in other plant species.

## Conclusions

In summary, the rice UbE3-ORFeome yeast library contains 98.94% of the whole E3 ligase genes. Utilizing this library, the known and new E3 ligases of four ubiquitinated proteins are efficiently identified. In addition, an uncharacterized F-Box E3 ligase as a hub interactor of the PAL family is identified, and the F-box protein degrades OsPALs to negatively regulate rice immunity. Thus, we demonstrate that the UbE3-ORFeome library is a powerful proteomic resource for the global identification of E3 ligases and the analysis of ubiquitination interactome and biological networks in plants. This approach also will benefit to other species in the analysis of protein–protein interactome maps and the establishment of ubiquitination interactome networks in eukaryotes.

## Methods

### Construction of the rice E3 ubiquitin ligase library

To obtain the full-length coding sequences (CDS) of rice E3 ligase genes, we germinated rice cultivar Nipponbare seeds on ½-strength Murashige and Skoog (½ MS) plates for ~ 1 week and transferred the seedlings to a greenhouse under 12/12 h light/dark conditions at a temperature of 20–30 °C. The leaves were collected from 3-week-old seedlings and frozen in liquid nitrogen for RNA extraction. To clone the rice E3 ligase genes into the pGADT7 vector, the CDS of each gene was amplified by PCR using rice cDNA, high-fidelity DNA polymerase, and gene-specific primers, and the cloned fragments were recombined into the pGADT7 vector via the *Eco* RI and *Bam* HI sites. Two bacterial transformants per clone were selected and verified by Sanger sequencing.

The genes that were not successfully PCR-amplified were synthesized using the following procedure. First, multiple primers (approximately 30~40 bp) with 10~15 bp overlapping sequences each other were chemically synthesized as fragments based on the gene coding sequence from RGAP 7. Second, the primer fragments were assembled into blocks of up to 800 bp of double-stranded DNA using PCR. If the gene size was over 800 bp, several blocks were then assembled and amplified once again using PCR to create one large double-stranded DNA block. Finally, the synthesized genes were cloned into the pGADT7 vector, and all the gene fragments were verified by Sanger sequencing.

### Yeast two-hybrid (Y2H) screening

Equal amounts of plasmid DNA harboring each E3 ligase gene were mixed well and transformed into yeast strain Y187. The yeast cells were spread on SD-Leu plates, cultured for 3 days, harvested, and mixed. Glycerol was added to the solution to a concentration of 15% for long-term storage at − 80 °C.

Before the screening, auto-activation of each pGBKT7-bait construct was determined by co-transforming yeast strain AH109 with the empty vector pGADT7. The lack of growth of a yeast culture on an SD-Leu-Trp-His-Ade plate indicated that no autoactivation occurred and the culture could be used for screening. We mated yeast strain AH109 carrying the pGBKT7-bait construct with strain Y187 containing plasmids with all the E3 ligase genes in the pGADT7 background at 30 °C with gentle shaking at 37 rpm for 20–24 h. The culture was checked under a microscope until a 3-lobed structure or a shape resembling “Mickey Mouse” appeared. Following centrifugation and re-suspension, the culture was spread onto SD-Leu-Trp-His-Ade plates and incubated at 30 °C for 3–10 days. The clones were picked, transferred to new SD-Leu-Trp-His-Ade plates, and incubated at 30 °C for 3 more days to further confirm the positive interaction. For PCR amplification, individual yeast clones were picked, transferred into ddH_2_O, and quickly lysed using liquid nitrogen. PCR was performed using lysed yeast as templates, the pGADT7 vector primers AD-F: CTATTCGATGATGAAGATACCCCACCAAACC and AD-R: GTGAACTTGCGGGGTTTTTCAGTATCTACGATT; the PCR products were purified and subjected to sequencing.

### Co-immunoprecipitation (Co-IP) assay

Co-IP assays were carried out by agro-infiltration of 4-week-old *N. benthamiana* leaves. Agrobacterium cultures carrying plasmids harboring GFP-tagged *OsSKIPa*, *OsNRPD1aC*, *NRR*, *rTGA2.1*, *OsPAL1*, and their cognate E3 ligase genes fused with HA tag were mixed and co-infiltrated into *N. benthamiana* leaves. Samples were collected at 72 h after agroinfiltration, and total protein was extracted as previously described [48]. Immunoprecipitation was performed with anti-GFP antibody (MBL, D153-11) or anti-HA antibody and protein G agarose beads. Immunoblotting was performed using an anti-GFP antibody (MBL, 598-7) or anti-HA antibody (MBL, M18907).

### Protein degradation assay in planta

Protein degradation experiments were performed via transient protein expression in *N. benthamiana* leaves [49, 50] and rice protoplasts. Agrobacterium cultures carrying plasmids harboring GFP-tagged *OsSKIPa*, *OsNRPD1aC*, *NRR*, *rTGA2.1*, *OsPAL1*, and their cognate E3 ligase genes fused with HA tag were mixed and co-infiltrated into *N. benthamiana* leaves. After 48 h, 50 μM MG132 (Millipore) or an equal volume of DMSO solution was infiltrated in the leaves, which were collected for protein extraction 24 h after MG132 treatment. For the protein degradation assay in rice protoplasts, plasmids harboring GFP-tagged *NRR* or *rTGA2.1* and their cognate E3 ligase genes fused with HA tag as well as the control GUS-HA construct were co-expressed in Nipponbare protoplasts. After 16 h, 50 μM MG132 or an equal volume of DMSO solution was added, and 4 h later, the protoplasts were collected for protein extraction. Protein abundance was detected by immunoblotting using anti-HA or anti-GFP antibody. ACTIN (Abmart, M20009L) or HSP was used as an internal protein control. *OsSKIPa*, *OsNRPD1aC*, or *OsPAL1* transcript levels were measured by RT-PCR, and *ACTIN* was used as the internal control.

### E3 ligase activity and ubiquitination assay in vitro

The full-length CDS of *OsRING77*, *OsRING113*, *OsRFPH2-10*, *P3IP1*, and *OsPUB46* were individually fused to the C-terminus of MBP in the pMal-C2X vector, the full-length OsRING116 was fused to the C-terminus of GST in the pGEX6p-1 vector, and the fusion proteins were expressed in *Escherichia coli* strain BL21. For RING-type E3 ligases, ubiquitination reaction mixtures containing E1 (wheat E1), E2 (AtUBC8), 2 μg/μL ubiquitin (U-100At, Boston Biochem, USA), and purified MBP-OsRING77, MBP-OsRING113, or MBP-OsRFPH2-10 were mixed in 1× reaction buffer (50 mM Tris-HCl, pH 7.4, 10 mM MgCl_2_, 5 mM ATP, and 2 mM DTT). The reactions were incubated at 30 °C for 2 h, and in vitro E3 ligase activity was determined using an anti-Ub antibody (Millipore, 05-944) and anti-MBP antibody (Abbkine, A02070-2) [51]. For U-box-type E3 ubiquitin ligase, purified MBP-P3IP1 or MBP-OsPUB46 was individually pre-incubated in total rice extracts prior to the assay [52]. For the substrate ubiquitination assay, equal amounts of purified GST-OsSKIPa or GST-OsNRPD1aC were added to the reaction mixture, and ubiquitination was measured using anti-Ub, anti-MBP, or anti-GST (BPI, AbM59001-2H5-PU) antibodies.

### Rice transformation and M. oryzae inoculation

The full-length coding fragments of *OsPAL1* and *OsPAL6* were amplified from the rice cultivar NPB and inserted into the pRHV-cGFP vector driven by the maize ubiquitin promoter to generate the *OsPAL1* and *OsPAL6* overexpression constructs [53]. The generated constructs were introduced into the calli of NPB via *Agrobacterium* tumefaciens-mediated transformation as described previously [54]. The *osfkb16* mutants were generated via CRISPR-Cas9 technology [55]. *OsPAL1* and *OsPAL6* overexpression lines were identified by quantitative real-time polymerase chain reaction (qRT-PCR) and the mutations in the *osfkb16* mutants were analyzed by sequencing.

For punch inoculation with *M. oryzae*, isolate RB22 was cultivated on an oat medium in darkness for 1 week at room temperature and then moved to light for spore induction. After 7–10 days, spore suspension (5 × 10^5^ spores/mL) of RB22 in 0.025% (v/v) Tween 20 was used for punch inoculation on the second leaf (from the top) of 6-week-old plants as previously described [56]. Disease symptoms on leaves were scored 14 days after inoculation. Relative fungal biomass was calculated by measuring the expression of the *M. oryzae MoPot2* with the DNA-based quantitative PCR assay.

### Accession numbers of the genes used in this study

The following are the accession numbers of the genes used in this study: OsFBX466 (LOC_Os02g38499), OsFBX55 (LOC_Os02g38589), OsFBO24 (LOC_Os12g05609), OsFBX481 (LOC_Os12g05709), OsRING62 (LOC_Os02g35347), OsRING66 (LOC_Os02g35365), OsRING202 (LOC_Os11g04280), OsRING203 (LOC_Os11g04281), OsRING77 (LOC_Os02g19140), OsRING113 (LOC_Os03g26370), OsRING171 (LOC_Os01g58400), OsRING394 (LOC_Os12g04590), OsRING199 (LOC_Os11g04680), OsFBX503 (LOC_Os07g17570), OsRING344 (LOC_Os08g42640), OsRING336 (LOC_Os05g41520), OsRING176 (LOC_Os01g58780), OsRING337 (LOC_Os09g12720), OsRING375 (LOC_Os04g22240), BTBT3 (LOC_Os11g37520), HBTB8 (LOC_Os12g08720), OsPUB28 (LOC_Os01g67500), OsPUB46 (LOC_Os04g34140), OsPUB49 (LOC_Os10g41220), OsPUB69 (LOC_Os08g13780), OsFBX68 (LOC_Os02g56810), OsFBX82 (LOC_Os03g20500), OsFBX389 (LOC_Os10g35920), BTBZ1 (LOC_Os01g66890), MBTB47 (LOC_Os10g29180), OsRING116 (LOC_Os01g38700), and OsFBK16 (LOC_Os06g39370).

## Supplementary Information

Additional file 1. Figure S1. Analysis of the ubiquitinated site-containing proteins in rice from published studies. Figure S2. Location of all E3 ligase encoding genes on the rice chromosome. Figure S3. Different types of ubiquitin E3 ligase-encoding genes in rice and the number of RT-PCR cloned and chemically synthesized E3 genes in this study. Figure S4. Confirmation of the interaction between OsUBC14 and its candidate E3s. Figure S5. E3 ubiquitin ligase activity of OsRING77, OsRING113, OsPUB28, OsPUB46, OsPUB49 and OsPUB69 *in vitro.* Figure S6. Ubiquitination assay of GST-OsSKIPa by MBP-OsPUB46. Figure S7. E3 ubiquitin ligase activity of OsRFPH2-10, P3IP1 and OsRING336 *in vitro*. Figure S8. The E3 ubiquitin ligase activity of OsRING116 *in vitro* and ubiquitination analysis of rTGA2.1. Figure S9. Transcript level of *OPAL1* in individual overexpression lines and the editing types of *OsFBK16.* Figure S10. Degradation assay of OsPAL5 and OsPAL6 by OsFBK16 *in vivo.* Figure S11. Transcript level of *OsPAL6* in individual overexpression lines.

Additional file 2. Table S1. Proteins containing ubiquitinated sites identified by four previous ubiquitome analyses.

Additional file 3. Table S2. Ubiquitinated substrates and their corresponding E3s.

Additional file 4. Table S3. Ubiquitin E3-encoding ORFs retaining full-length CDS.

Additional file 5. Table S4. Expression analysis of all E3 ligase encoding genes.

Additional file 6. Table S5. E3-type interactors screened from the rice UbE3 library.

Additional file 7. Review history.

## Data Availability

All data generated or analyzed during this study are included in this published article and its supplementary information files. The rice UbE3 yeast two-hybrid library is available under NCBI with the BioProject ID: PRJNA841249 [57].
