# Supplementary material for: An ORFeome of rice E3 ubiquitin ligases for global analysis of the ubiquitination interactome
Source: Genome Biol. 2022 Jul 11;23:154. doi: 10.1186/s13059-022-02717-8 (PMC9277809; doi:10.1186/s13059-022-02717-8)
Supplement: Supplementary file 1 — Additional file 1. Figure S1. Analysis of the ubiquitinated site-containing proteins in rice from published studies. Figure S2. Location of all E3 ligase encoding genes on the rice chromosome. Figure S3. Different types of ubiquitin E3 ligase-encoding genes in rice and the number of RT-PCR cloned and chemically synthesized E3 genes in this study. Figure S4. Confirmation of the interaction between OsUBC14 and its candidate E3s. Figure S5. E3 ubiquitin ligase activity of OsRING77, OsRING113, OsPUB28, OsPUB46, OsPUB49 and OsPUB69 in vitro. Figure S6. Ubiquitination assay of GST-OsSKIPa by MBP-OsPUB46. Figure S7. E3 ubiquitin ligase activity of OsRFPH2-10, P3IP1 and OsRING336 in vitro. Figure S8. The E3 ubiquitin ligase activity of OsRING116 in vitro and ubiquitination analysis of rTGA2.1. Figure S9. Transcript level of OPAL1 in individual overexpression lines and the editing types of OsFBK16. Figure S10. Degradation assay of OsPAL5 and OsPAL6 by OsFBK16 in vivo. Figure S11. Transcript level of OsPAL6 in individual overexpression lines. [file 13059_2022_2717_MOESM1_ESM.ppt]

## Slide 1
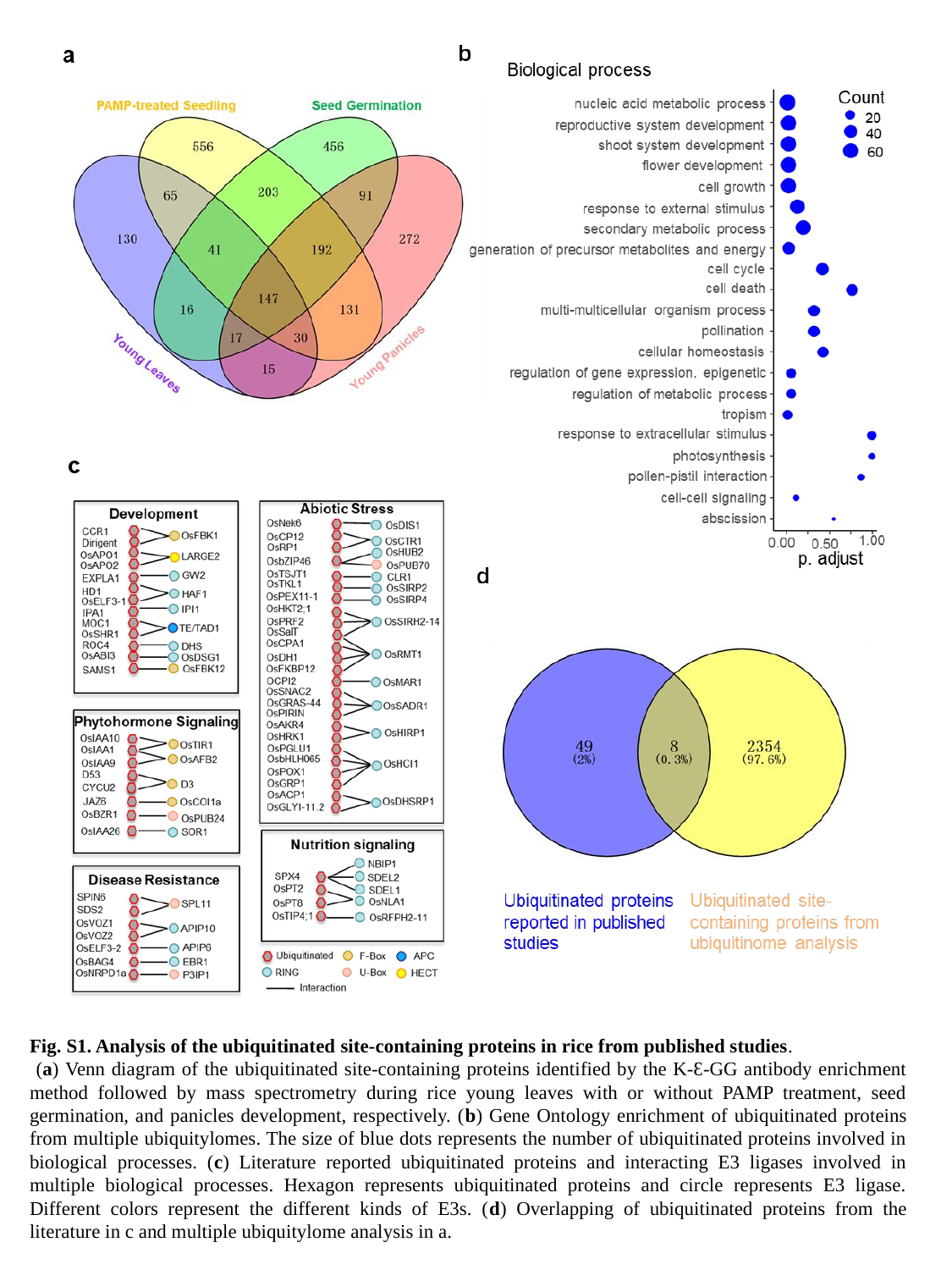

Fig. S1. Analysis of the ubiquitinated site-containing proteins in rice from published studies.
 (a) Venn diagram of the ubiquitinated site-containing proteins identified by the K-Ɛ-GG antibody enrichment method followed by mass spectrometry during rice young leaves with or without PAMP treatment, seed germination, and panicles development, respectively. (b) Gene Ontology enrichment of ubiquitinated proteins from multiple ubiquitylomes. The size of blue dots represents the number of ubiquitinated proteins involved in biological processes. (c) Literature reported ubiquitinated proteins and interacting E3 ligases involved in multiple biological processes. Hexagon represents ubiquitinated proteins and circle represents E3 ligase. Different colors represent the different kinds of E3s. (d) Overlapping of ubiquitinated proteins from the literature in c and multiple ubiquitylome analysis in a.

## Slide 2
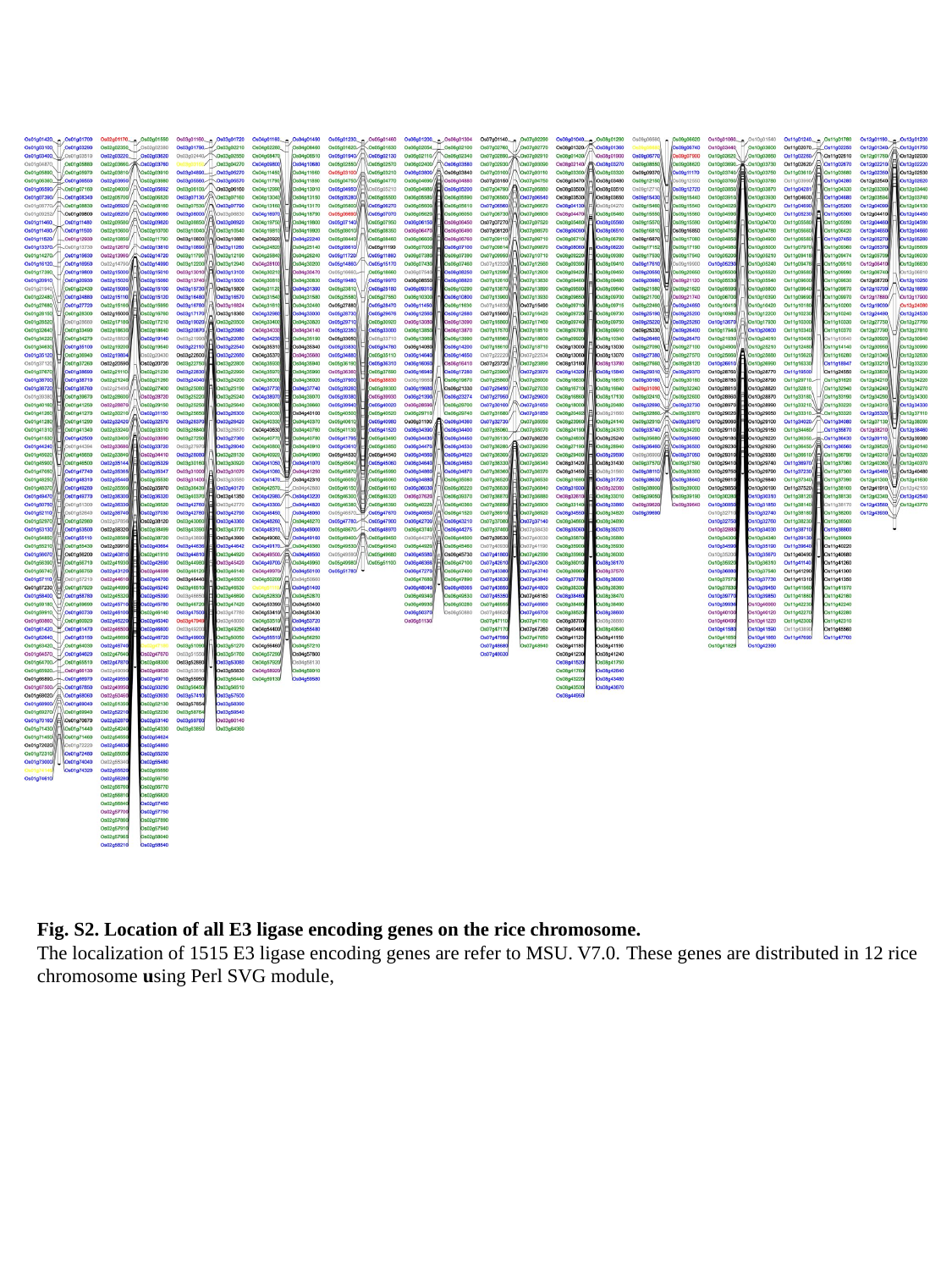

Fig. S2. Location of all E3 ligase encoding genes on the rice chromosome.
The localization of 1515 E3 ligase encoding genes are refer to MSU. V7.0. These genes are distributed in 12 rice chromosome using Perl SVG module,

## Slide 3
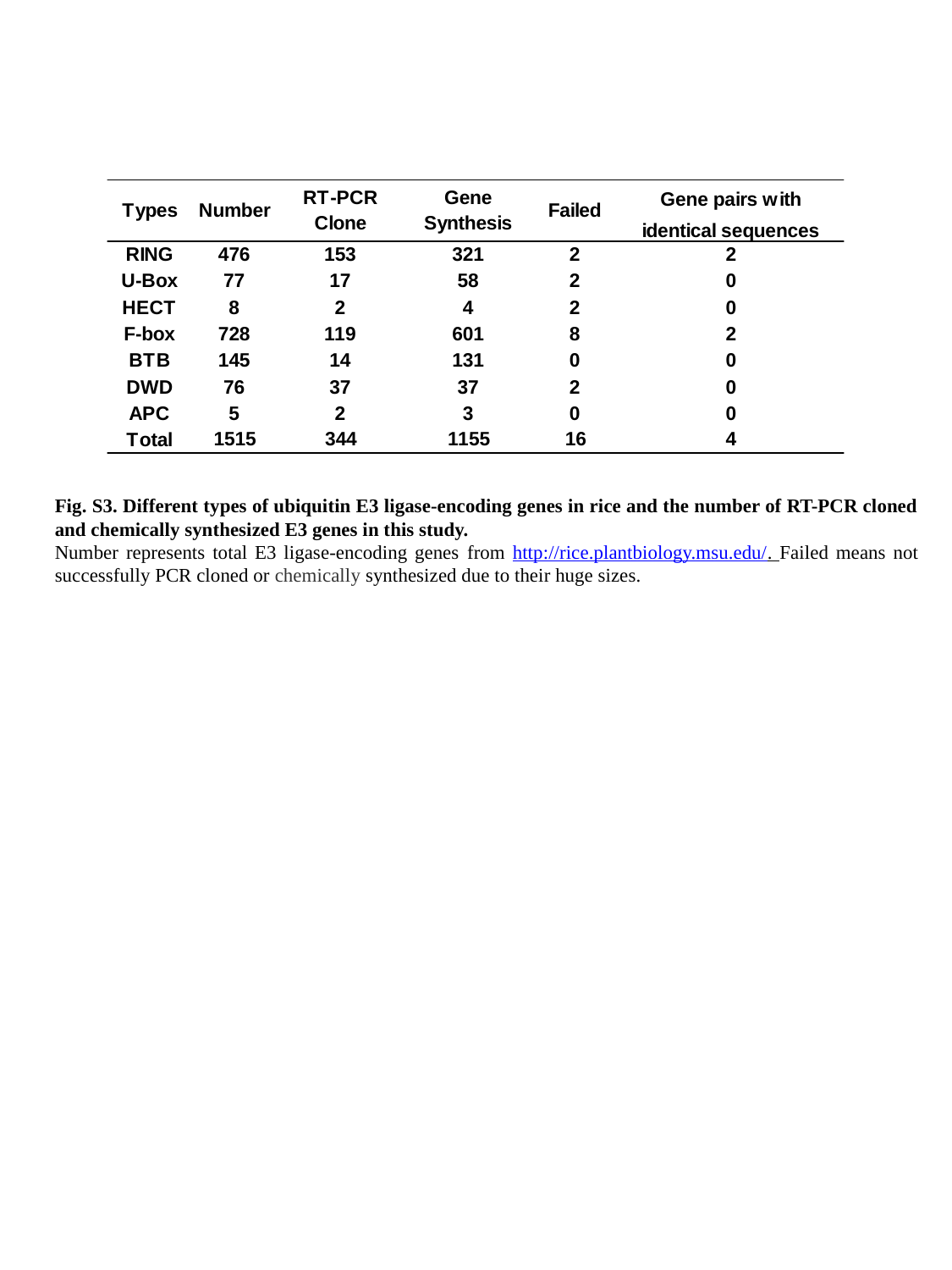

Fig. S3. Different types of ubiquitin E3 ligase-encoding genes in rice and the number of RT-PCR cloned and chemically synthesized E3 genes in this study.
Number represents total E3 ligase-encoding genes from http://rice.plantbiology.msu.edu/. Failed means not successfully PCR cloned or chemically synthesized due to their huge sizes.

## Slide 4
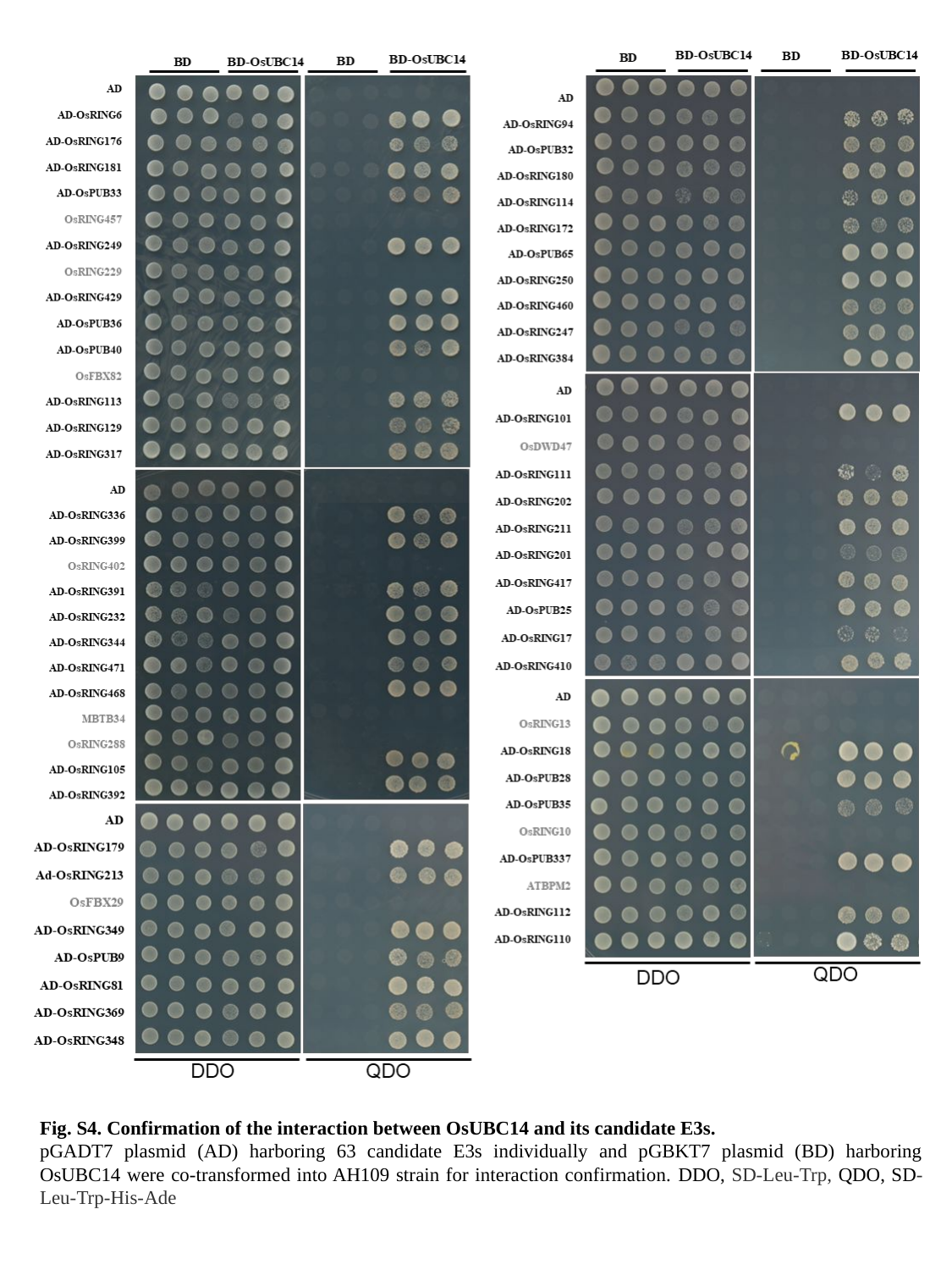

Fig. S4. Confirmation of the interaction between OsUBC14 and its candidate E3s.
pGADT7 plasmid (AD) harboring 63 candidate E3s individually and pGBKT7 plasmid (BD) harboring OsUBC14 were co-transformed into AH109 strain for interaction confirmation. DDO, SD-Leu-Trp, QDO, SD-Leu-Trp-His-Ade

## Slide 5
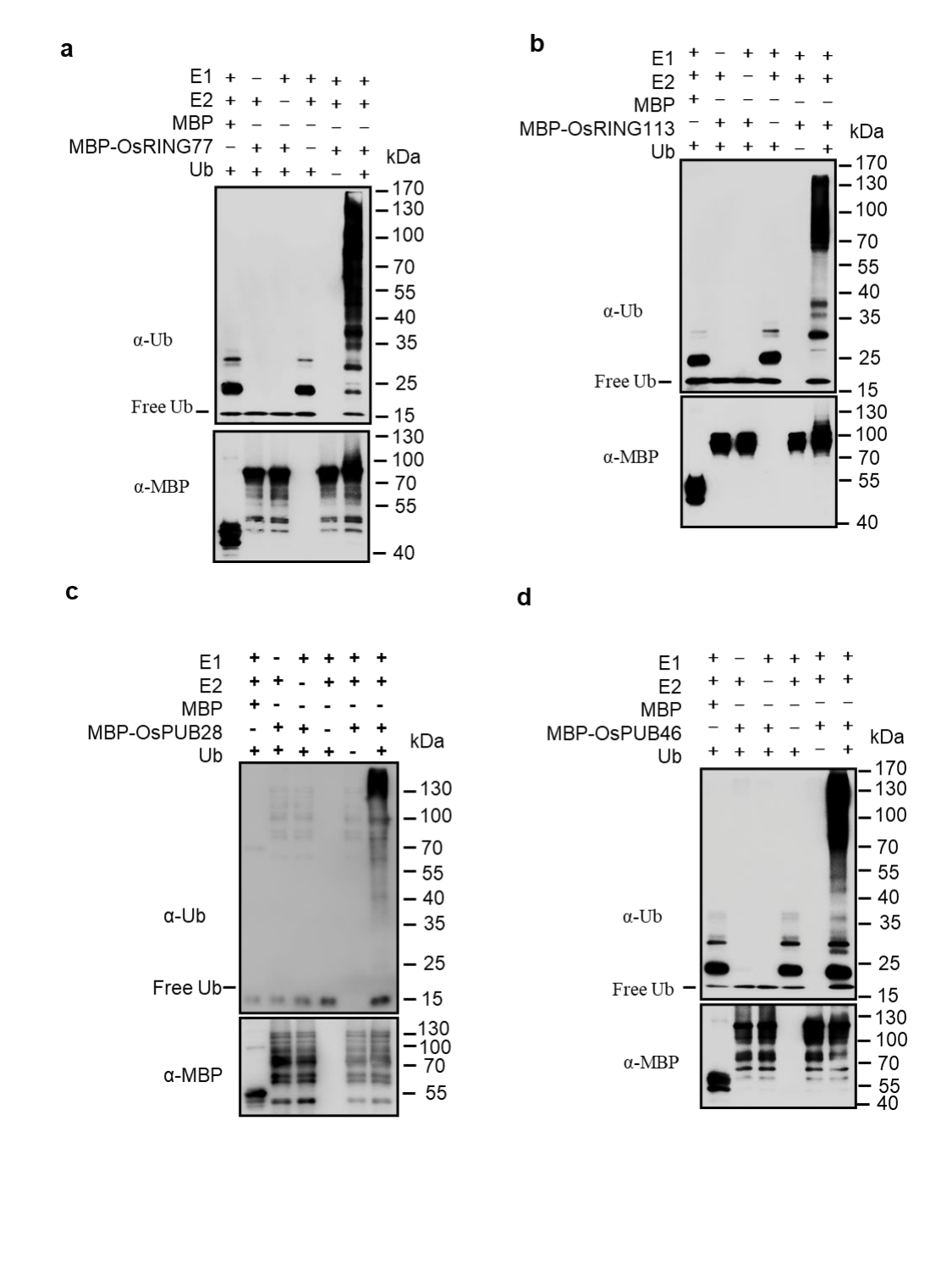

## Slide 6
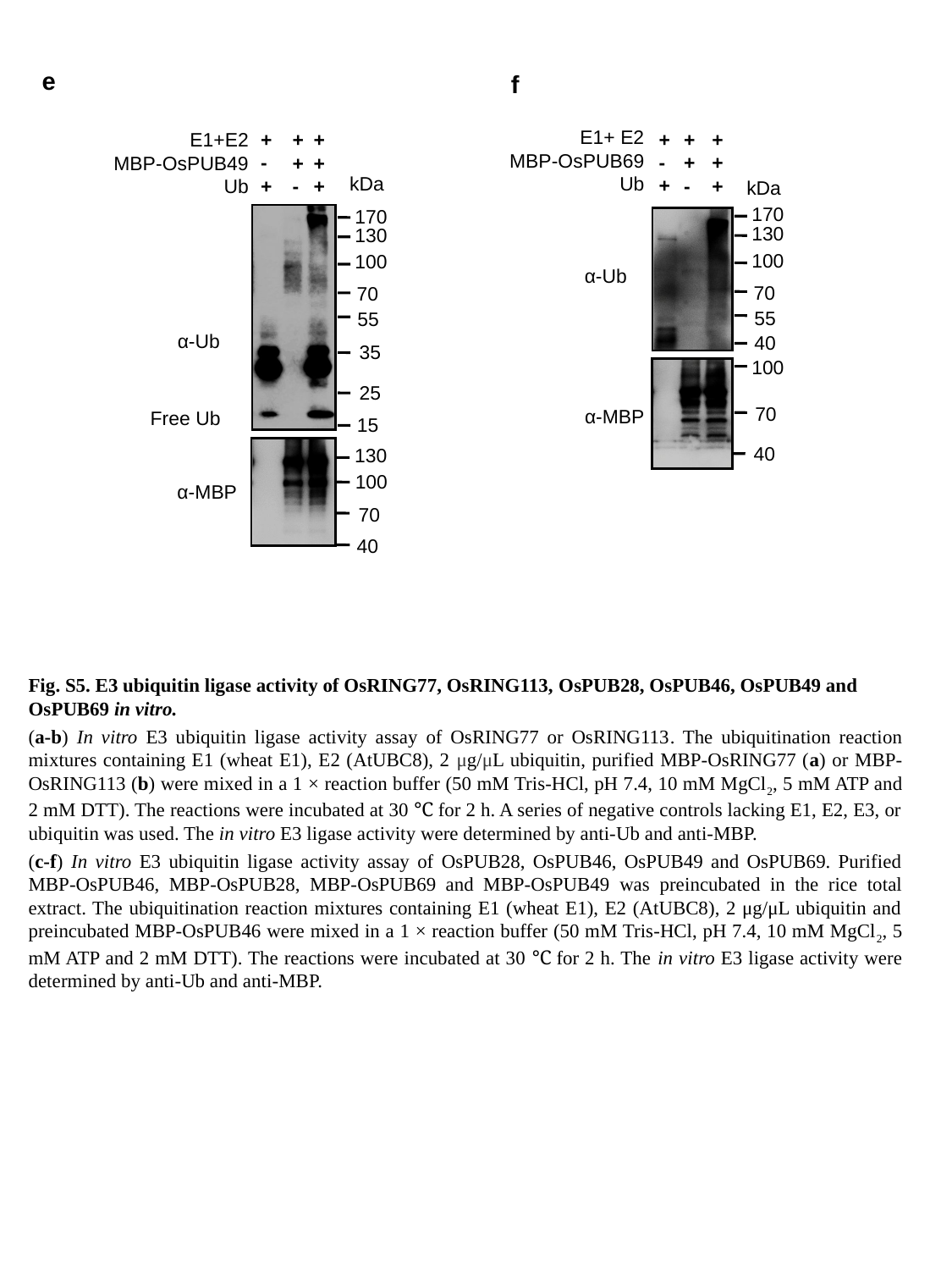

e
f
E1+ E2
MBP-OsPUB69
Ub
+
-
+
+
-
+
+
+
-
+
+
+
kDa
170
130
100
70
55
α-Ub
35
25
Free Ub
15
130
100
α-MBP
70
40
E1+E2
MBP-OsPUB49
Ub
+
+
-
+
+
+
kDa
170
130
100
α-Ub
70
55
40
100
70
α-MBP
40
Fig. S5. E3 ubiquitin ligase activity of OsRING77, OsRING113, OsPUB28, OsPUB46, OsPUB49 and OsPUB69 in vitro.
(a-b) In vitro E3 ubiquitin ligase activity assay of OsRING77 or OsRING113. The ubiquitination reaction mixtures containing E1 (wheat E1), E2 (AtUBC8), 2 μg/μL ubiquitin, purified MBP-OsRING77 (a) or MBP-OsRING113 (b) were mixed in a 1 × reaction buffer (50 mM Tris-HCl, pH 7.4, 10 mM MgCl2, 5 mM ATP and 2 mM DTT). The reactions were incubated at 30 ℃ for 2 h. A series of negative controls lacking E1, E2, E3, or ubiquitin was used. The in vitro E3 ligase activity were determined by anti-Ub and anti-MBP.
(c-f) In vitro E3 ubiquitin ligase activity assay of OsPUB28, OsPUB46, OsPUB49 and OsPUB69. Purified MBP-OsPUB46, MBP-OsPUB28, MBP-OsPUB69 and MBP-OsPUB49 was preincubated in the rice total extract. The ubiquitination reaction mixtures containing E1 (wheat E1), E2 (AtUBC8), 2 μg/μL ubiquitin and preincubated MBP-OsPUB46 were mixed in a 1 × reaction buffer (50 mM Tris-HCl, pH 7.4, 10 mM MgCl2, 5 mM ATP and 2 mM DTT). The reactions were incubated at 30 ℃ for 2 h. The in vitro E3 ligase activity were determined by anti-Ub and anti-MBP.

## Slide 7
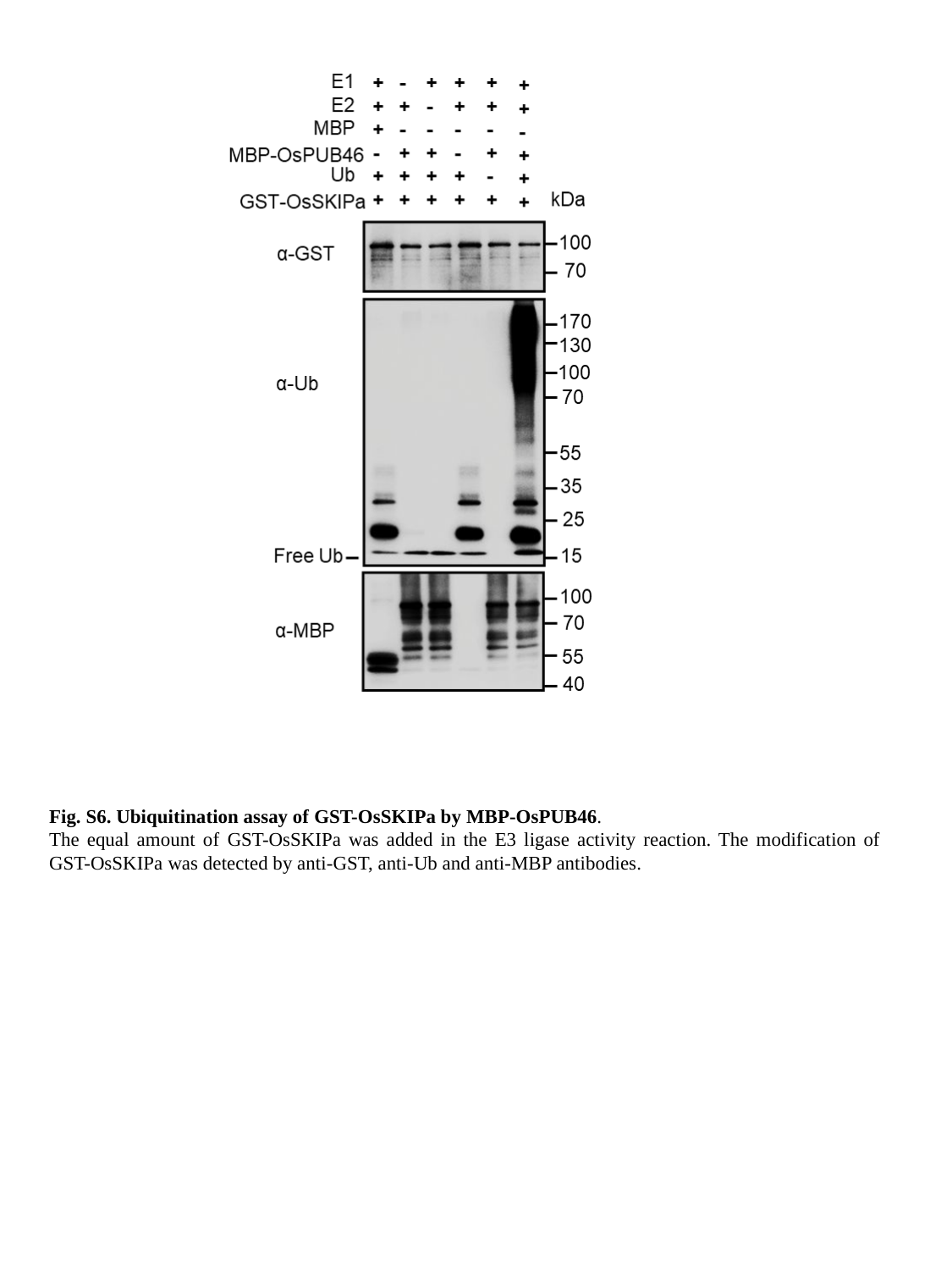

Fig. S6. Ubiquitination assay of GST-OsSKIPa by MBP-OsPUB46.
The equal amount of GST-OsSKIPa was added in the E3 ligase activity reaction. The modification of GST-OsSKIPa was detected by anti-GST, anti-Ub and anti-MBP antibodies.

## Slide 8
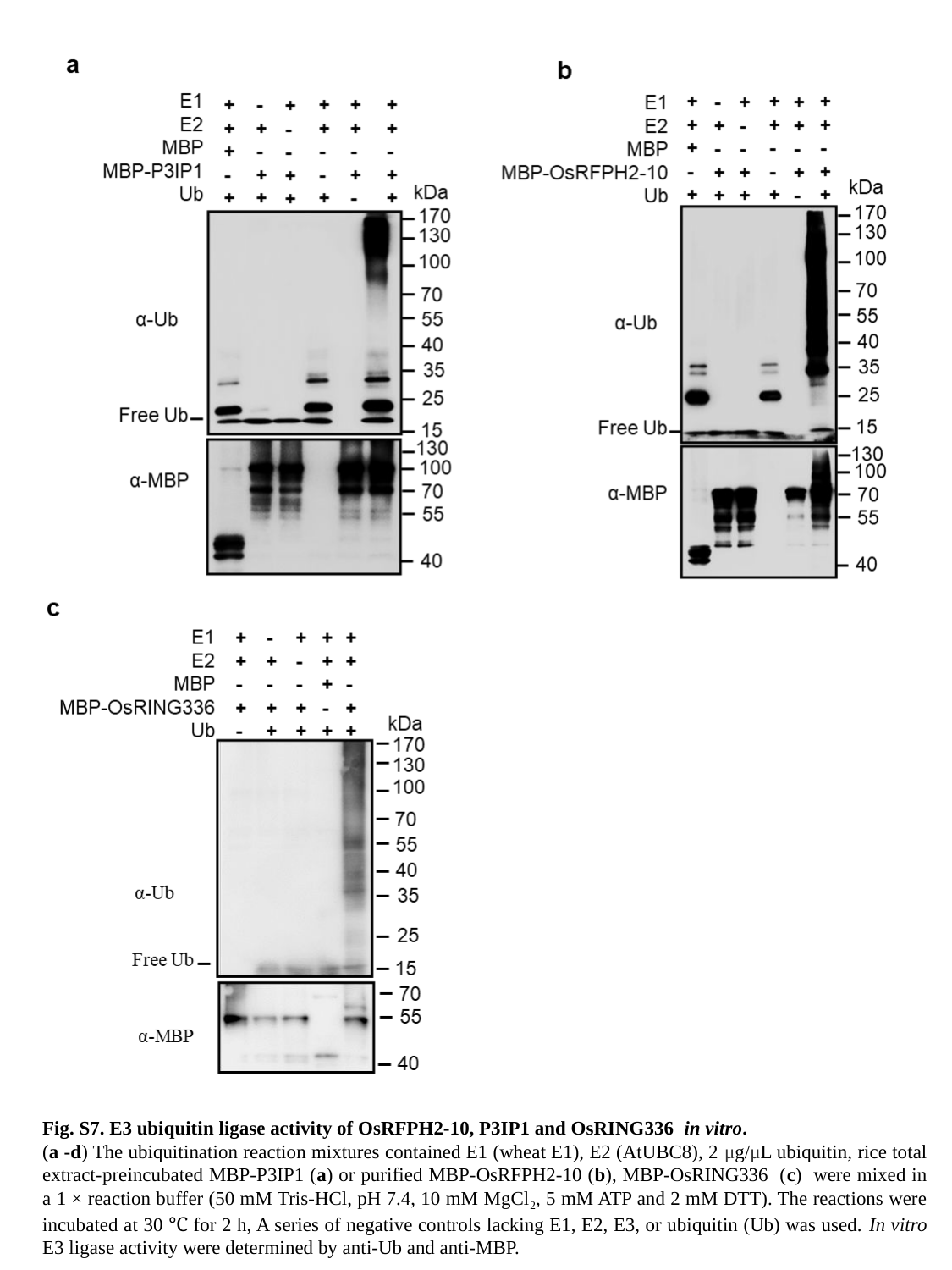

Fig. S7. E3 ubiquitin ligase activity of OsRFPH2-10, P3IP1 and OsRING336 in vitro.
(a -d) The ubiquitination reaction mixtures contained E1 (wheat E1), E2 (AtUBC8), 2 μg/μL ubiquitin, rice total extract-preincubated MBP-P3IP1 (a) or purified MBP-OsRFPH2-10 (b), MBP-OsRING336 (c) were mixed in a 1 × reaction buffer (50 mM Tris-HCl, pH 7.4, 10 mM MgCl2, 5 mM ATP and 2 mM DTT). The reactions were incubated at 30 ℃ for 2 h, A series of negative controls lacking E1, E2, E3, or ubiquitin (Ub) was used. In vitro E3 ligase activity were determined by anti-Ub and anti-MBP.

## Slide 9
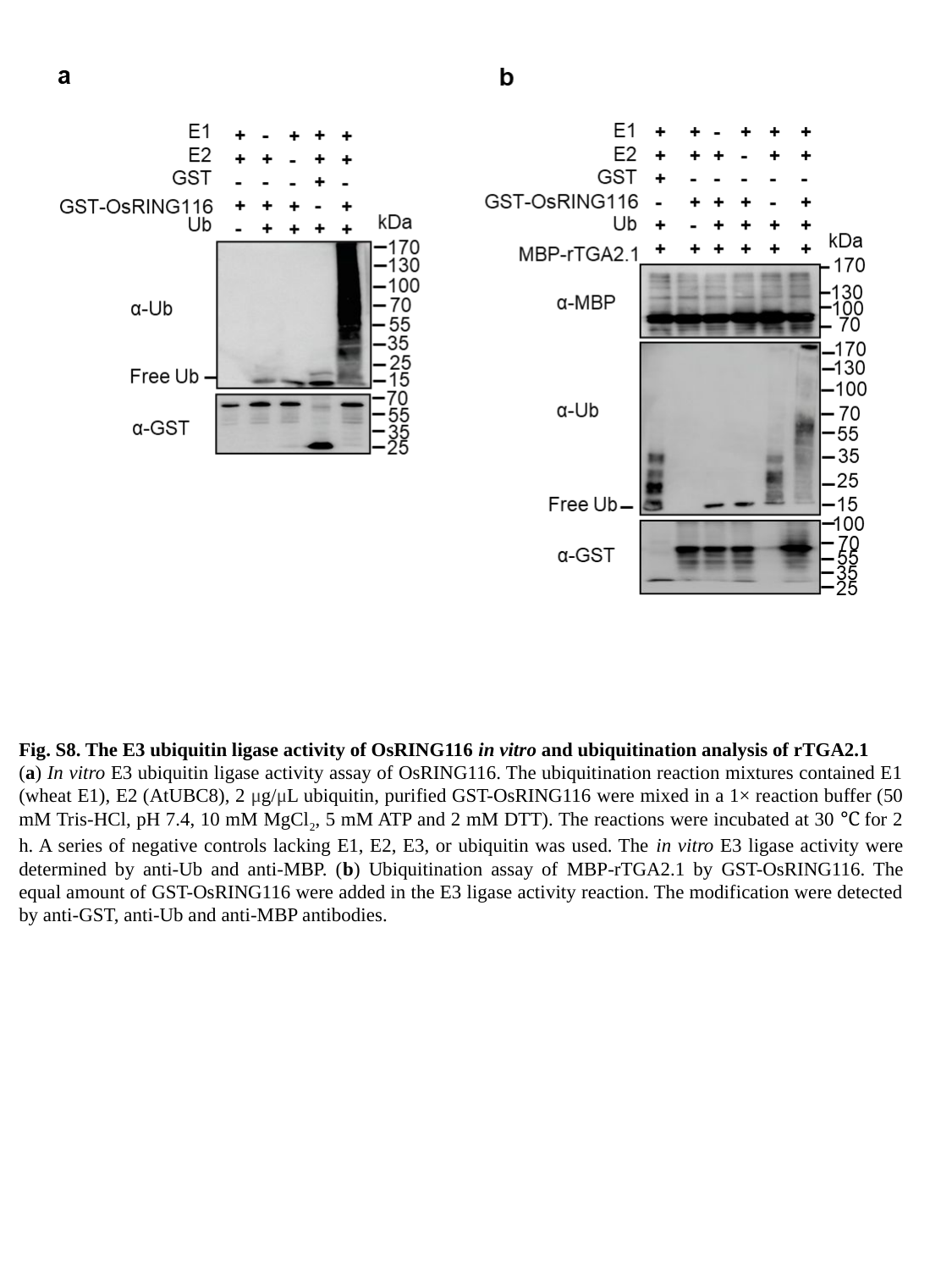

Fig. S8. The E3 ubiquitin ligase activity of OsRING116 in vitro and ubiquitination analysis of rTGA2.1
(a) In vitro E3 ubiquitin ligase activity assay of OsRING116. The ubiquitination reaction mixtures contained E1 (wheat E1), E2 (AtUBC8), 2 μg/μL ubiquitin, purified GST-OsRING116 were mixed in a 1× reaction buffer (50 mM Tris-HCl, pH 7.4, 10 mM MgCl2, 5 mM ATP and 2 mM DTT). The reactions were incubated at 30 ℃ for 2 h. A series of negative controls lacking E1, E2, E3, or ubiquitin was used. The in vitro E3 ligase activity were determined by anti-Ub and anti-MBP. (b) Ubiquitination assay of MBP-rTGA2.1 by GST-OsRING116. The equal amount of GST-OsRING116 were added in the E3 ligase activity reaction. The modification were detected by anti-GST, anti-Ub and anti-MBP antibodies.

## Slide 10
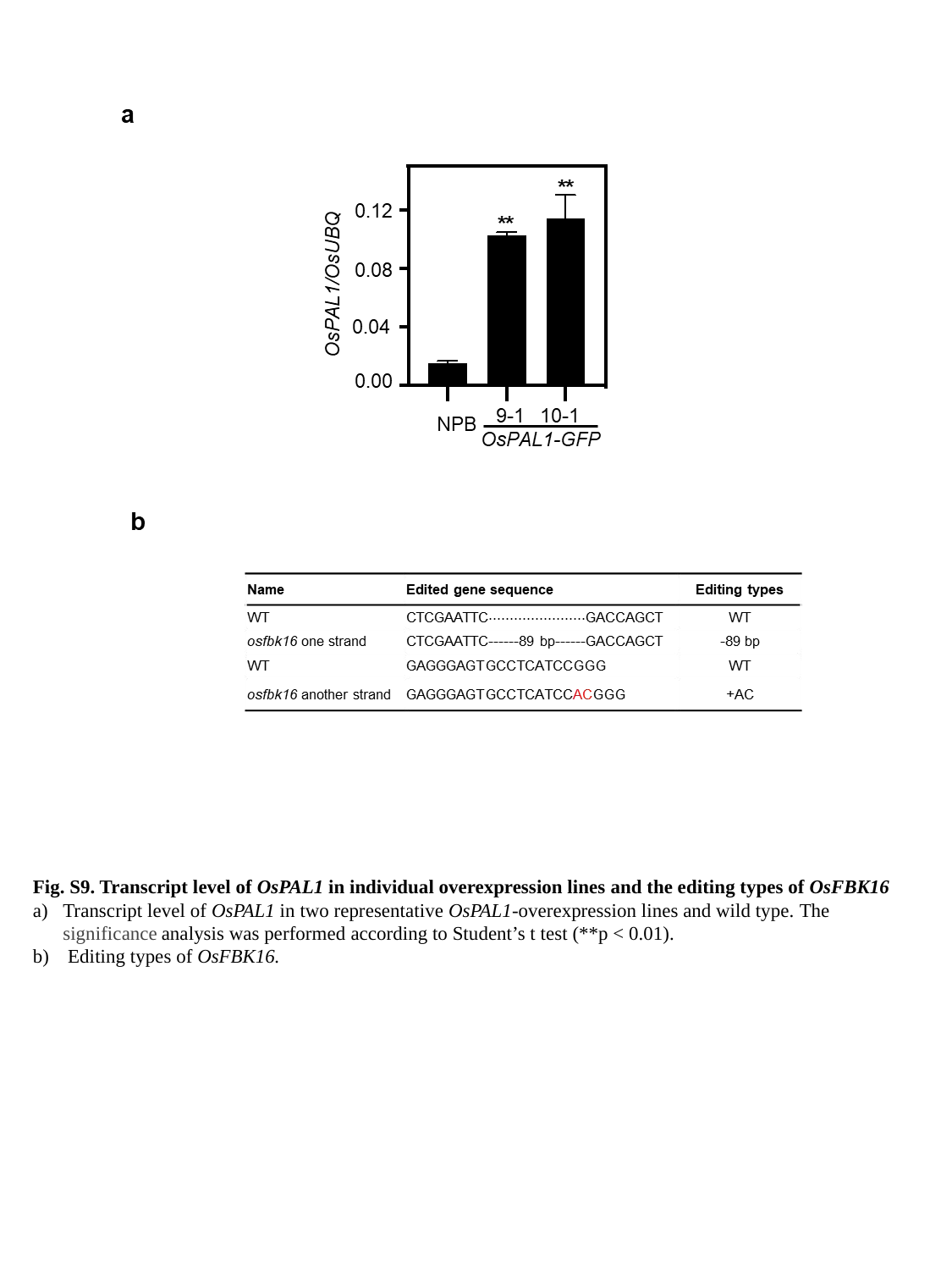

Fig. S9. Transcript level of OsPAL1 in individual overexpression lines and the editing types of OsFBK16
Transcript level of OsPAL1 in two representative OsPAL1-overexpression lines and wild type. The significance analysis was performed according to Student’s t test (**p < 0.01).
 Editing types of OsFBK16.

## Slide 11
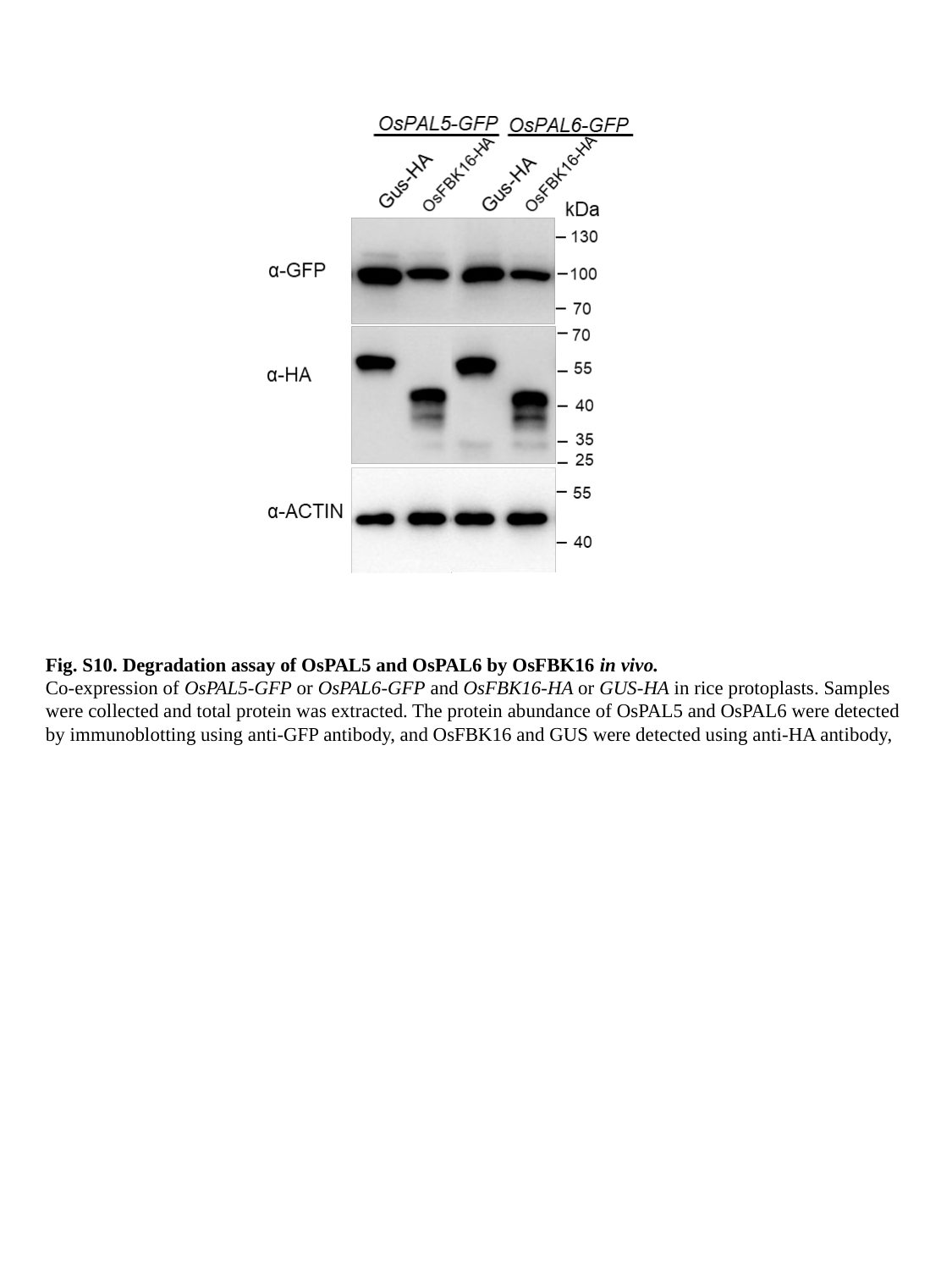

Fig. S10. Degradation assay of OsPAL5 and OsPAL6 by OsFBK16 in vivo.
Co-expression of OsPAL5-GFP or OsPAL6-GFP and OsFBK16-HA or GUS-HA in rice protoplasts. Samples were collected and total protein was extracted. The protein abundance of OsPAL5 and OsPAL6 were detected by immunoblotting using anti-GFP antibody, and OsFBK16 and GUS were detected using anti-HA antibody,

## Slide 12
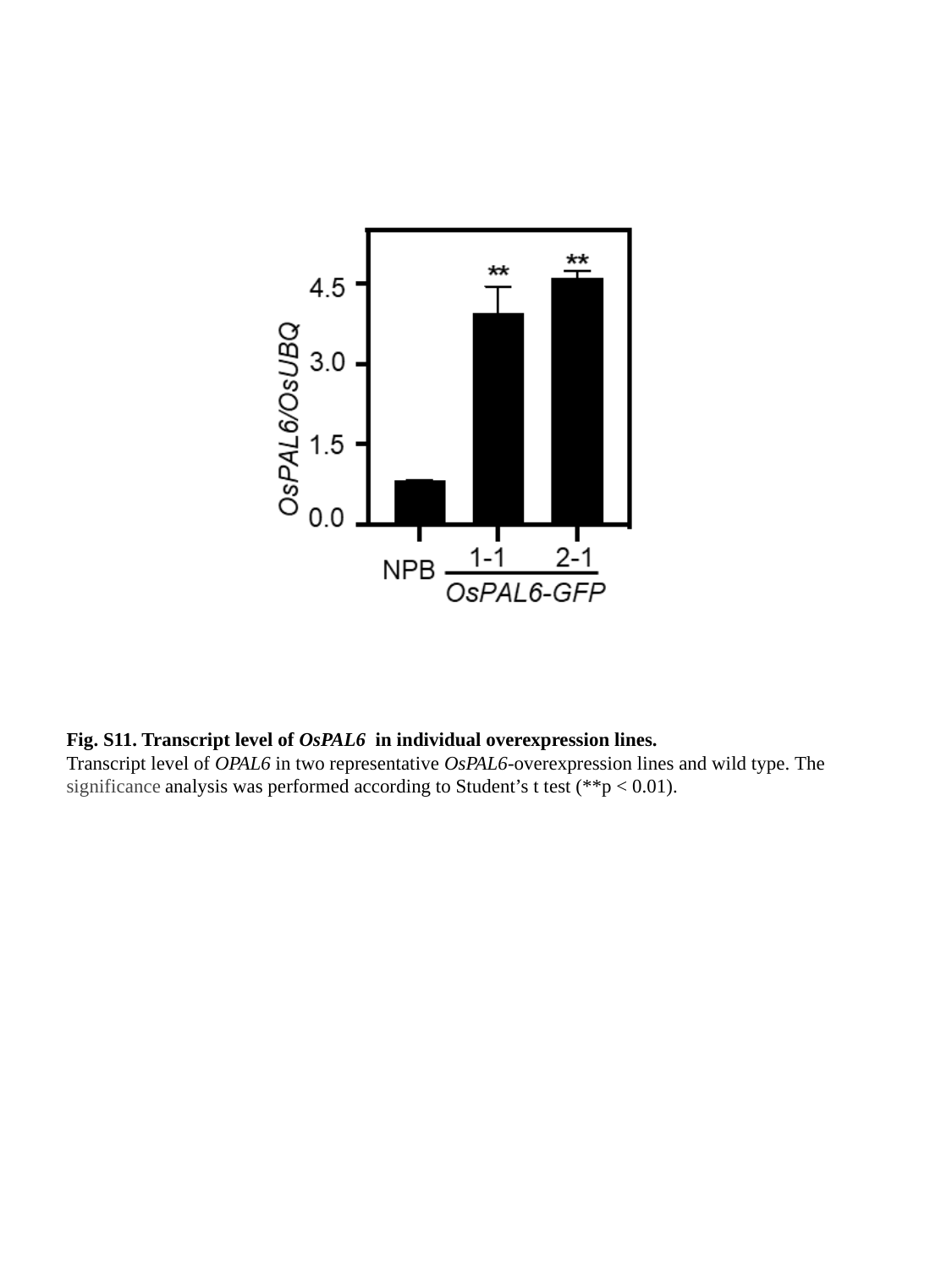

Fig. S11. Transcript level of OsPAL6 in individual overexpression lines.
Transcript level of OPAL6 in two representative OsPAL6-overexpression lines and wild type. The significance analysis was performed according to Student’s t test (**p < 0.01).
