# Supplementary material for: An ORFeome of rice E3 ubiquitin ligases for global analysis of the ubiquitination interactome
Source: Genome Biol. 2022 Jul 11;23:154. doi: 10.1186/s13059-022-02717-8 (PMC9277809; doi:10.1186/s13059-022-02717-8)
Supplement: Supplementary file 7 — Additional file 7. Review history. [file 13059_2022_2717_MOESM7_ESM.docx]

Review History

**First round of review**

**Reviewer 1**

**Were you able to assess all statistics in the manuscript, including the appropriateness of statistical tests used?**

There are no statistics in the manuscript.

**Comments to author:**

Protein ubiquitylation play very important roles in mediating protein turn over and recycling, and hence involved in many biological processes. Deciphering the mechanism of protein ubiquitylation is of great importance, of which characterization of E3 ligase is the priority because of its substrate specificity. In this study, the authors constructed a large scale E3 screening library through cloning and synthesizing the annotated E3 genes. Furthermore, they conducted a series of screening and verification experiments. The whole study and its obtained data are valuable. I still have some concerns listed below:

1，I would like suggest the authors to draw a map to show the location of all these E3 ligase encoding genes on the chromosome.

2, Figure 1C, I would like suggest to show the exact number rather than the percentage of each type of E3 genes.

3, For sure, it is ok to synthesize the rest of E3 encoding genes based on their sequence. But the authors should think about the possibility of the existence of pseudogenes. Although it is unnecessary to clone them again, it is better to show the expression of these E3 genes based on the existence RNA-seq data.

4, As we known, E3 ligase could mediate the degradation of its target protein, because of which overexpression of some of E3 genes will be lethal to yeast. I am curious that the authors did not mention this at all. Does this mean that all the transformant yeasts grew well?

5, substrate specificity is the typical feature of E3 ligase. Then how to explain that one substrate could be recognize by multi-E3s?

6, one concern is that both BTB-type and F-box type E3 include multi-protein components. Without other components, they may not be able to catalyze the ubiquitilation of their targets. This is true for BTB-type, but why only F-box E3 could catalyze the ubiquitylation of PALs in the transient system?

7, Since the authors have obtained the transgenic rice for OsFBK16, it will be good to check the degradation of PALs in this transgenic line, which should be more convincible for their data.

**Reviewer 2**

**Were you able to assess all statistics in the manuscript, including the appropriateness of statistical tests used?**

There are no statistics in the manuscript.

**Comments to author:**

In this manuscript, the authors took a huge effort to construct a yeast two-hybrid prey ORFeome library for 1,499 ubiquitin E3 ligase members in rice, including mono-subunit E3 genes and substrate adaptors of multi-subunit E3 complexes. They then selected four known E3 substrates, OsSKIPa, OsNPRD1a, NRR/rTGA2.1 and OsPALs, to test the effectiveness of this nearly complete ubiquitin E3 ligase-encoding (UbE3) library in finding the pairwise interactions between substrates and the cognate E3s. While this reviewer appreciates the authors' effort and motivation in tackling the challenge in the field that lacks an effective approach for high throughput identification of E3-substrate network, the work presented in this manuscript did not find a solid solution either due to several major concerns listed below.

1. In the 1,499 clones, the authors are not sure whether all of them are bona fide E3 members. Bioinformatic predictions only provide some statistical evidence showing how likely the product of a protein coding gene is an E3 member. Whether the product has the biochemical activity of an E3 should be further experimentally varified. Thus, this library most likely will yield many false positive clones, in which the encoded products do not have the E3 activity. The failure in detecting the E3 activities of OsPUB46 in targeting OsSKIPa degradation and the NRR/rTGA2.1 degradation by their several interacting E3 members demonstrated this problem.

2. Although the library is constructed based on a nearly complete set of E3 ORFeome, I also found the resulting data could complicate the biological interpretation. It is well known that the E3 ligase group is largely expanded in plants and many of its members are under rapid evolution with very low expression, which the authors also recognized. Therefore, even if a novel E3 ligase is identified for a substrate by Y2H screening, it may have nothing to do with the substrate in vivo due to extremely low expression or even no expression at all.

3. Since all interaction partners identified through Y2H needs to be further confirmed by degradation and ubiquitylation assays, I don't see much difference between a regular Y2H cDNA library and this ORFeome library. Instead, much more effective Y2H-based protein-protein interaction approaches have been developed recently, for example, CrY2H-seq. Therefore, I only see that this UbE3 library may have some incremental but not much significant impacts to the field.

4. In addition to the above weakness of the manuscript, the authors did not present their work logically nor comprehensively. For example, I don't see any connection about the gene ontology analysis of ubiquitinated proteins with the construction of UbE3. For the test screens on four known E3 substrates, the authors did not examine the activities of all interacting partners. I don't know whether this is because of the lack of E3 activities of these interacting partners. I also found that only testing few E3 substrates is not significant either because a similar work can be done on a regular Y2H library by a graduate or even an undergraduate student.

**Authors Response**

**Point-by-point responses to the reviewers’ comments:**

Reviewer #1:

Protein ubiquitylation play very important roles in mediating protein turn over and recycling, and hence involved in many biological processes. Deciphering the mechanism of protein ubiquitylation is of great importance, of which characterization of E3 ligase is the priority because of its substrate specificity. In this study, the authors constructed a large scale E3 screening library through cloning and synthesizing the annotated E3 genes. Furthermore, they conducted a series of screening and verification experiments. The whole study and its obtained data are valuable.

*Response：Thanks for the nice comments on our manuscript.*

I still have some concerns listed below: 1. I would like suggest the authors to draw a map to show the location of all these E3 ligase encoding genes on the chromosome.

*Response：Thanks for the good suggestion. We obtained the physical location of 1515 E3 genes from the rice genomic database (MSU, version 7.0) and used the Perl SVG module to analyze the distribution of these genes on 12 rice chromosomes. We added the data in Additional file Fig. S2 and relevant descriptions on page 7, lines 193-194.*

2. Figure 1C, I would like suggest to show the exact number rather than the percentage of each type of E3 genes.

*Response：We changed the percentage of E3 genes to the exact number of E3 genes in Fig. 1c.*

3. For sure, it is ok to synthesize the rest of E3 encoding genes based on their sequence. But the authors should think about the possibility of the existence of pseudogenes. Although it is unnecessary to clone them again, it is better to show the expression of these E3 genes based on the existence RNA-seq data. *Response：We agree with the suggestion and searched the Plant Public RNA-seq Database (PPRD, http://ipf.sustech.edu.cn/pub/plantrna/) to check the expression of the 1515 E3 ligase genes. PPRD contains 11,726 rice RNA-seq libraries is a searchable database (Plant Biotechnol J 2022. doi.org/10.1111/pbi.13798). The results revealed that all the 1515 E3 genes were expressed in rice. We added the data in Additional file 5, Table S4 and relevant descriptions on page 7, lines 205-210.*

4. As we known, E3 ligase could mediate the degradation of its target protein, because of which overexpression of some of E3 genes will be lethal to yeast. I am curious that the authors did not mention this at all. Does this mean that all the transformant yeasts grew well?

*Response：We did not test if overexpression of each E3 gene causes cell death or not in yeast. Instead, we cloned all the E3 genes into pGADT7 one by one, and mixed an equal amount of plasmid DNA of the constructs and transformed the mixed DNA into yeast strain Y187. Thus, we could not exclude that few clones can induce lethality in yeast.*

5. substrate specificity is the typical feature of E3 ligase. Then how to explain that one substrate could be recognize by multi-E3s?

*Response： Substrate specificity means one E3 only targets one or few substrates. The possible reasons of one substrate could be recognize by multi-E3s are: (1) one substrate has multiple functions in different cellular compartments. For example, in mammalian cells, the E3 ligase SCFSkp2 degrades cyclin-dependent kinase inhibitor (CKI) P21 to regulate the cell cycle (Nakayama et al. 2004, Dev. Cell 6, 661–672), while the other E3 ligase CRL2LRR1 degrades the cytoplasmic P21 to control the actin cytoskeleton and motility (Starostina et al. 2010, Dev. Cell 19, 753–764). Moreover, MKRN1 (E3 Makorin RING Finger Protein 1) degrades P21 to control apoptosis (Lee, 2009, EMBO J. 28, 2100–2113). (2) some substrates have spatial and temporalspecific expression, which may be degraded by different E3s. For example, the E3 ligase CRL4Cdt2 degrades P21 in S phase (Kim et al. 2008, Genes Dev. 22, 2507– 2519), while the closely related RING E3 MDM2 and MDMX degrade P21 in G1 and early S phase (Jin et al. 2003, EMBO J. 22, 6365–6377, Jin. et al. 2008, Mol. Cell. Biol.) (3) some substrates can be recognized and degraded by homologous E3 ligases. For example, two Arabidopsis U-box type E3 ubiquitin ligases, PUB25 and PUB26, polyubiquitinate MYB15, leading to MYB15 degradation and thus enhanced CBF expression under cold stress (Wang et al., Dev Cell 2019;51(2):222-235.e5.)*

6. one concern is that both BTB-type and F-box type E3 include multi-protein components. Without other components, they may not be able to catalyze the ubiquitilation of their targets. This is true for BTB-type, but why only F-box E3 could catalyze the ubiquitylation of PALs in the transient system?

*Response：Thanks for your question. Agroinfiltration in Nicotiana benthamiana leaves is an efficient protein degradation assay for detecting ubiquitination in vivo (Plant J, 2010, 61 (5) :893-903). It is true that BTB-type and F-box type E3 are not able to catalyze the ubiquitination of their targets without other components like SKP1, CUL1 and Rbx1. Actually, N. benthamiana leaves contain conserved SCF complex components including NbSKP1, NbCUL1, and NbRbx1 as well as E1 and E2, and has been used for plant protein degradation assays. For example, in Arabidopsis, the degradation of the transcription factor BES1 by F-box E3 ubiquitin ligase BAF1 (Wang et al., 2021, Plant Cell), and the degradation of the ZTL protein by F-box protein SDC (Tian et al., 2021). I added these two papers in the manuscript reference on page 19, line 561.*

7. Since the authors have obtained the transgenic rice for OsFBK16, it will be good to check the degradation of PALs in this transgenic line, which should be more convincible for their data.

*Response：This is a good suggestion. Although we have the osfbk16 mutant, the PAL antibodies are not available, thus we cannot directly detect the degradation of PAL in rice plants. In order to confirm the degradation in rice cells, we co-expressed OsFBK16 and OsPAL5 or OsPAL6 in rice protoplasts and detected the degradation of the two PAL proteins. The results showed that OsFBK16 promoted the degradation of OsPAL5 and OsPAL6 in rice cells. We added the new degradation results in Additional File 1: Fig. S10 and relevant descriptions on page 14, lines 402-406.*

Reviewer #2:

In this manuscript, the authors took a huge effort to construct a yeast two-hybrid prey ORFeome library for 1,499 ubiquitin E3 ligase members in rice, including mono-subunit E3 genes and substrate adaptors of multi-subunit E3 complexes. They then selected four known E3 substrates, OsSKIPa, OsNPRD1a, NRR/rTGA2.1 and OsPALs, to test the effectiveness of this nearly complete ubiquitin E3 ligase-encoding (UbE3) library in finding the pairwise interactions between substrates and the cognate E3s. While this reviewer appreciates the authors' effort and motivation in tackling the challenge in the field that lacks an effective approach for high throughput identification of E3-substrate network, the work presented in this manuscript did not find a solid solution either due to several major concerns listed below.

1. In the 1,499 clones, the authors are not sure whether all of them are bona fide E3 members. Bioinformatic predictions only provide some statistical evidence showing how likely the product of a protein coding gene is an E3 member. Whether the product has the biochemical activity of an E3 should be further experimentally varified. Thus, this library most likely will yield many false positive clones, in which the encoded products do not have the E3 activity. The failure in detecting the E3 activities of OsPUB46 in targeting OsSKIPa degradation and the NRR/rTGA2.1 degradation by their several interacting E3 members demonstrated this problem.

*Response：Thanks for your comments. The main purpose of this study is to establish a proteomic platform for the community to screen for candidate E3 ligases of interested substrates. Once a candidate E3 is identified, the lab can perform more molecular analysis such as E3 ligase activity assay and functional characterizations. As you may know, it is easy to detect the ligase activity of single-subunit E3 ligases like RING and U-box E3s, but it is still a technical challenge to detect the ligase activity of multiple-subunit E3s in vitro. It will be a huge project to test the E3 ligase activity of all 553 single-subunit E3s (476 RING-type and 77 U-box-type) because it takes a lot of time to optimize the expression conditions for each clone, the activity of some E3s needs additional modification in vivo, like phosphorylation and the cost for all the reagents used in the ligase assay for 553 clones is beyond the resource of a regular laboratory.*

*To address your concerns, we performed in vitro protein ubiquitination experiments for the OsSKIPa-interacting E3s, and demonstrated that OsRING77, OsRING113, OsPUB46, OsPUB28 OsPUB69 and OsPUB49 exhibited strong E3 ligase activity while OsRING171 and OsPUB58 did not. Although OsPUB46 showed strong E3 ligase activity (Additional file 1：Fig S5d), it did not degrade OsSKIPa (Additional file 1：Fig S6); We added these new data in Additional file 1: Fig. S5c, d, f and relevant descriptions on page 10, lines 268-271.*

*For the OsNRPD1a-interacting E3s, we performed in vitro protein ubiquitination experiments and demonstrated that OsRFPH2-10, OsRING336 and P3IP1 exhibited strong E3 ligase activity, while OsPUB61 did not. We added the new data in Additional file 1: Fig. S7c and relevant descriptions on page 11, lines 309-312.*

*For the NRR interacting E3s, we only identified one single subunit E3 ligase, OsPUB69, which exhibited strong E3 ligase activity (Additional file 1：Fig S5f) but did not promote degradation of NRR.*

*For rTGA2.1-interacting E3s, we only identified one single subunit E3 ligase OsRING116, which showed strong E3 ligase activity but did not promote degradation of rTGA2.1 (Additional file 1：Fig S8a-b). The relevant descriptions are added on page 11, lines 357-359.*

*Besides these four known substrates, we have collaborated with dozens of research groups to identify E3 ligases of their interested proteins using our UbE3 library. Up to now, more than 100 E3 ligases have been identified. Among them, 33 are singlesubunit E3s. We tested E3 ligase activity of all clones and found that 29 showed strong E3 ligase activity. Three E3s did not exhibit any activity. The other E3 was not expressed well in E. coli. Based these results, we conclude that more than 80% of the examined single-subunit E3s have E3 ligase activity. Following are the E3 ligase activity data for your references.*

2. Although the library is constructed based on a nearly complete set of E3 ORFeome, I also found the resulting data could complicate the biological interpretation. It is well known that the E3 ligase group is largely expanded in plants and many of its members are under rapid evolution with very low expression, which the authors also recognized. Therefore, even if a novel E3 ligase is identified for a substrate by Y2H screening, it may have nothing to do with the substrate in vivo due to extremely low expression or even no expression at all.

*Response：Thanks for your comments. We agree that E3 ligase number is expanded in plants compared to that in mammals, but it doesn’t mean that many E3 members are not expressed. The reason that only a small proportion of the genes were successfully cloned using PCR method is due to low abundance of the transcripts in rice seedlings. Alternatively, perhaps the complex structures of some genes inhibited the amplification of the full-length ORFs. We searched the Plant Public RNA-seq Database (PPRD, http://ipf.sustech.edu.cn/pub/plantrna/) to check the expression of the 1515 E3 ligase genes. PPRD contains 11,726 rice RNA-seq libraries is a searchable database (Plant Biotechnol J 2022. doi.org/10.1111/pbi.13798). The results revealed that all the 1515 E3 genes were expressed in rice. We added the data in Additional file 5, Table S4 and relevant descriptions on page 7, lines 205-210.*

3. Since all interaction partners identified through Y2H needs to be further confirmed by degradation and ubiquitylation assays, I don't see much difference between a regular Y2H cDNA library and this ORFeome library. Instead, much more effective Y2H-based protein-protein interaction approaches have been developed recently, for example, CrY2H-seq. Therefore, I only see that this UbE3 library may have some incremental but not much significant impacts to the field.

*Response: This UbE3 ORFeome library provides more than 98% coverage of rice E3s. Through one library screen, one researcher can identify all E3s that interact with the interested substrate within 2 weeks. As you know, some E3 genes are only expressed in specific tissues or developmental stages or under some stress conditions. It is impossible to clone all rice E3 genes in a conventional cDNA library. Moreover, in the conventional cDNA libraries, two-thirds of the clones are not amenable to fusion in frame with the N-terminal GAL4 activation domain that is required for validating assays. In contrary, the UbE3 library contains equal concentrations of the E3 plasmids that allows us to identify all possible E3s of a substrate. Moreover, all the clones in the UbE3 library were sequencing-verified, thus guaranteeing the correct translation of the clones in yeast. The library was transformed into yeast strain Y187, which can easily be mated with a bait yeast strain. To the best of our knowledge, this is the first complete E3 ligase library constructed in plants. Thus, our UbE3 library will be a great proteomic resource for the rice community.*

4. In addition to the above weakness of the manuscript, the authors did not present their work logically nor comprehensively. For example, I don't see any connection about the gene ontology analysis of ubiquitinated proteins with the construction of UbE3. For the test screens on four known E3 substrates, the authors did not examine the activities of all interacting partners. I don't know whether this is because of the lack of E3 activities of these interacting partners. I also found that only testing few E3 substrates is not significant either because a similar work can be done on a regular Y2H library by a graduate or even an undergraduate student.

*Response：Thanks for your comments. We performed gene ontology analysis of the ubiquitinated proteins from multiple ubiquitylome projects to figure out which kind of signaling pathways are affected by ubiquitination. Our analysis showed that ubiquitinated proteins may play critical roles in plant multiple signaling pathways including growth, development as well as stress response. Pls see the data in Additional file 1：Fig S1b and we added relevant descriptions on page 6, lines 165- 167.*

*According to your suggestion, we examined the E3 ligase activity of all 13 singlesubunit type-E3s but not for the multiple-subunit type-E3s because a reliable assay is still unavailable for this type of E3s. The results showed that 10 of the tested 13 E3s showed E3 ligase activity, indicating that most of the clones in our library are functional. As mentioned above, our library has over 98% coverage of the E3 ligase genes and a graduate or undergraduate student can finish a substrate screen within 2 weeks. Based on the published results and our screening experience in this study, a substrate can be targeted by multiple E3s. Even though we can get 1-2 E3 clones from a conventional cDNA library, we may miss many clones that are not included in the cDNA library or because the frequency of these clones is too low.*

**Second round of review**

**Reviewer 1**

As noticed in the submission form, this is a re-submitted manuscript, which was subjected to reviewing by two reviewers including me. I just read through the manuscript carefully along with my previous concerns. I agree that the authors have addressed most of my concerns, and their new data and explanations are convincible to me.

One more concern is still about the cloning of the E3 ligase encoding genes. Since the authors extracted the RNAs just from leaves of young seedlings, I prefer to believe that the main reason is because of the tissue-specific expression of the genes. I suggest the authors to discuss this point in combination of the RNA-seq data.

**Reviewer 2**

In this resubmission, the authors added several new data that have certainly improved their work. I appreciate their effort in testing autoubiquitination activity of 33 new single-subunit E3 ligases. While I would like to reserve my original opinion arguing that the scientific significance of this work is yet low, I do recognize that the authors' effort was enormous. This UbE3 yeast two-hybrid library could be a good resource for the rice research community. However, before I am completely convinced by the authors' argument about their resource/material contribution, I want to know the accessibility of this library. The current version has not clearly addressed where the UbE3 yeast two-hybrid library has be deposited. Since the major contribution/merit of this work is the construction of this library, the authors need to make sure the material is accessible to the community. A repository of the library and/or accessibility should be clearly described in "Availability of data and materials".

**Authors Response**

**Point-by-point responses to the reviewers’ comments:**

Reviewer #1:

As noticed in the submission form, this is a re-submitted manuscript, which was subjected to reviewing by two reviewers including me. I just read through the manuscript carefully along with my previous concerns. I agree that the authors have addressed most of my concerns, and their new data and explanations are convincible to me. One more concern is still about the cloning of the E3 ligase encoding genes. Since the authors extracted the RNAs just from leaves of young seedlings, I prefer to believe that the main reason is because of the tissue-specific expression of the genes. I suggest the authors to discuss this point in combination of the RNA-seq data.

*Response: Thanks for your comments. We added the sentences in discussion: All of the E3 genes were expressed in the Plant Public RNA-seq Database, while only 22.71% of them were cloned using RT-PCR method in our experimental conditions, the small proportion is mainly because of the tissue-specific expression of E3s in leaves. in page 18, lines 422-425.*

Reviewer #2:

In this resubmission, the authors added several new data that have certainly improved their work. I appreciate their effort in testing autoubiquitination activity of 33 new single-subunit E3 ligases. While I would like to reserve my original opinion arguing that the scientific significance of this work is yet low, I do recognize that the authors' effort was enormous. This UbE3 yeast two-hybrid library could be a good resource for the rice research community. However, before I am completely convinced by the authors' argument about their resource/material contribution, I want to know the accessibility of this library. The current version has not clearly addressed where the UbE3 yeast two-hybrid library has be deposited. Since the major contribution/merit of this work is the construction of this library, the authors need to make sure the material is accessible to the community. A repository of the library and/or accessibility should be clearly described in "Availability of data and materials".

*Response: Thanks for your comments. We have submitted the UbE3 yeast two-hybrid library to the NCBI, with the BioProject accession PRJNA841249 and Submission ID: SUB11506479. Researchers can* access *the library information through the link : https://www.ncbi.nlm.nih.gov/bioproject/PRJNA841249. We added “The rice UbE3 yeast two-hybrid library is available under NCBI with the BioProject ID: PRJNA841249” in “Availability of data and materials” in page 23, lines 666-667.*
